# Bioprospection of Natural Sources of Polyphenols with Therapeutic Potential for Redox-Related Diseases

**DOI:** 10.3390/antiox9090789

**Published:** 2020-08-26

**Authors:** Regina Menezes, Alexandre Foito, Carolina Jardim, Inês Costa, Gonçalo Garcia, Rita Rosado-Ramos, Sabine Freitag, Colin James Alexander, Tiago Fleming Outeiro, Derek Stewart, Cláudia N. Santos

**Affiliations:** 1 CEDOC, Chronic Diseases Research Centre, NOVA Medical School/Faculdade de Ciências Médicas, Universidade NOVA de Lisboa, Campo dos Mártires da Pátria, 130, 1169-056 Lisboa, Portugal; regina.menezes@nms.unl.pt (R.M.); rita.ramos@nms.unl.pt (R.R.-R.); 2 iBET, Instituto de Biologia Experimental e Tecnológica, Apartado 12, 2781-901 Oeiras, Portugal; cjardim@medicina.ulisboa.pt (C.J.); inescosta.bms@gmail.com (I.C.); ggarcia@campus.ul.pt (G.G.); 3 Instituto de Tecnologia Química e Biológica, Universidade Nova de Lisboa, Av. da República, 2780-157 Oeiras, Portugal; 4 Environmental and Biochemical Science Group, The James Hutton Institute, Dundee DD2 5DA, UK; alex.foito@hutton.ac.uk (A.F.); Sabine.Freitag@hutton.ac.uk (S.F.); Derek.Stewart@hutton.ac.uk (D.S.); 5 Biomathematics and Statistics Scotland, Invergowrie, Dundee DD2 5DA, UK; Colin.Alexander@hutton.ac.uk; 6 Department of Experimental Neurodegeneration, Center for Biostructural Imaging of Neurodegeneration, University Medical Center Goettinge, 37073 Göttingen, Germany; touteir@gwdg.de; 7 Max Planck Institute for Experimental Medicine, 37075 Göttingen, Germany; 8 Translational and Clinical Research Institute, Faculty of Medical Sciences, Newcastle University, Framlington Place, Newcastle Upon Tyne NE2 4HH, UK; 9 School of Engineering and Physical Sciences, Institute of Mechanical, Process and Energy Engineering, Heriot-Watt University, Edinburgh EH14 4AS, UK

**Keywords:** bioactivity-based assays, cyanidin, metabolomics, *Rubus* genus, (poly)phenols, yeast-based discovery platform

## Abstract

Plants are a reservoir of high-value molecules with underexplored biomedical applications. With the aim of identifying novel health-promoting attributes in underexplored natural sources, we scrutinized the diversity of (poly)phenols present within the berries of selected germplasm from cultivated, wild, and underutilized *Rubus* species. Our strategy combined the application of metabolomics, statistical analysis, and evaluation of (poly)phenols’ bioactivity using a yeast-based discovery platform. We identified species as sources of (poly)phenols interfering with pathological processes associated with redox-related diseases, particularly, amyotrophic lateral sclerosis, cancer, and inflammation. In silico prediction of putative bioactives suggested cyanidin–hexoside as an anti-inflammatory molecule which was validated in yeast and mammalian cells. Moreover, cellular assays revealed that the cyanidin moiety was responsible for the anti-inflammatory properties of cyanidin–hexoside. Our findings unveiled novel (poly)phenolic bioactivities and illustrated the power of our integrative approach for the identification of dietary (poly)phenols with potential biomedical applications.

## 1. Introduction

Plants synthesize a staggering variety of secondary metabolites that provide a chemically diverse pool of high-value small molecules with potential application for human health that cannot be matched by any synthetic libraries [1]. The use of plants in traditional medicine dates back to antiquity and is still, despite huge investments into combinatorial chemistry and high-throughput screens, an important source of novel drugs and metabolites with a myriad of underexplored pharmacological and biotechnological applications [2]. From conventional folk medicine to the scientific validation of protective properties, (poly)phenolic compounds have been implicated and/or identified as underpinning the beneficial health properties of several plants.

*Rubus* is a large and diverse genus of the Rosaceae family comprising more than 250 species with the most commonly known being red and black raspberries and blackberries. These fruits are characterized by their high polyphenolic content and diversity, which make them a major source of (poly)phenols with potential importance for human health. This includes redox-related diseases, such as neurodegeneration and cancer, which is consistent with the well-described role of (poly)phenols in targeting signaling pathways regulating redox homeostasis. Besides sharing oxidative stress and chronic inflammation as common pathological processes, neurodegenerative diseases (NDs) are also known as conformational disorders, as they are associated with protein misfolding and aggregation [3,4] in a process thought to lead to neuronal death. Alzheimer’s disease (AD) pathology is associated to the accumulation of Aβ42 amyloid plaques [5] and hyperphosphorylated tau neurofibrillary tangles [6]. The accumulation of concentric hyaline cytoplasmic inclusions of α-synuclein (αSyn), known as Lewy bodies (LBs), is the major pathological hallmark of Parkinson’s disease (PD) and other LB diseases [7,8]. Proteotoxic aggregates in neuronal cells of Huntington’s disease (HD) patients are formed by N-terminal polyglutamine (polyQ)-expanded huntingtin (HTT) [9]. In amyotrophic lateral sclerosis (ALS), fused in sarcoma or translocated in liposarcoma (FUS/TLS) protein has been implicated in the formation of toxic aggregates and neuronal demise [10,11].

With the goal of harnessing the diversity of *Rubus* (poly)phenols for the discovery of new phenolic compounds of value, we developed an integrative approach that combined the power of metabolomics, for polyphenolic content characterization, and a Simple Molecular Architecture Research Tool (SMART) discovery platform for filtering potential bioactivities to be further explored in advanced pre-clinical models [12,13,14]. The platform is composed of yeast strains expressing human disease genes associated with the most-studied NDs as cited before (*Aβ42* [15], *SNCA* [16], *HTTpQ103* [17], *FUS*/*TLS* [18]), cancer (*RAS* and *RAF* [19]), and inflammation (*CRZ1* [20], the yeast orthologue of human Nuclear Factor of Activated T-cell—NFAT). This is possible due to the high degree of evolutionary conservation of fundamental biological processes among eukaryotes, which has established the budding yeast as a powerful model for the identification of molecular targets amenable for therapeutic intervention and lead molecules with health-promoting potential [21,22]. Benefiting from the easy and low-cost handling, facile genetic manipulation and the possibility to search against specific molecular targets, yeast-based screening technologies have proved to be very useful for the identification of promising drug candidates [12,13,23,24,25] including the flavonoids quercetin and epigallocatechin gallate [26].

## 2. Materials and Methods

### 2.1. Plant Material and Extraction Procedure

A range of different cultivars and species from the *Rubus* genus cultivated in Portugal (Odemira) and UK (Dundee) (Table S1) were manually harvested in the field at full ripeness as assessed by picker. Samples were kept in a cool box until they were transferred to −20 °C storage. Samples were extracted as described by Dudnik et al. [12]. In summary, approximately 50 g of frozen fruit from each species/cultivar was weighted and transferred into a solvent-proof blender containing 150 mL of pre-cooled 50 ng/mL Morin (Sigma–Aldrich, Gillingham, UK) solution prepared with 0.2% formic acid methanolic solution. Samples were then blended with three pulses of 10 s duration and subsequently filtered using Whatman filter paper grade 1. The filtrate was aliquoted and solvent-dried using a speed-vac (VWR, Lutterworth, UK) and subsequently lyophilized. Dried extracts were flushed with N_2_ and stored at −20 °C until analysis by Liquid Chromatography coupled to a Time-of-Flight Mass Spectrometer (LC-ToF-MS). The filtrates to be used in the cell assays were solvent-dried using speed-vac, resuspended in CH_3_COOH/H_2_O (50/50), and subjected to solid-phase extraction [27].

### 2.2. Total Phenolic Quantification

Total phenolic content of the eluates were determined using the Folin–Ciocalteu method adapted to a microplate reader [27]. The eluates were aliquoted, freeze-dried, and frozen at −20 °C.

### 2.3. Phenolic Profile Determination by LC-ToF-MS

Analysis of sample extracts was performed as described by Dudnik et al. [12]. Briefly, dried extracts from each species/cultivar were resolubilized in triplicate using 2 mL of a 75% methanol solution with 0.1% formic acid. Five hundred microliters of the extract were decanted into filter vials, sealed with 0.45 mm Polytetrafluoroethylene-lined screwcap (Thomson Instrument Company, London, UK) and transferred into the autosampler. The analysis was achieved using an Agilent LC-ToF-MS system consisting of a quaternary pump (Agilent 1260, Cheadle, UK), a diode-array-detector (DAD) (Agilent 1260), a temperature control device (Agilent 1260), and a thermostat (Agilent 1290) coupled to an Agilent 6224 time-of-flight (ToF) instrument. Five microliters of the sample were injected onto a 2 × 150 mm (4 µm) C18 column (Phenomenex, Torrance, CA, USA) fitted with a C18 4 × 2 mm Security Guard™ cartridge (Phenomenex, Torrance, CA, USA). Sample and column temperature were maintained at 4 °C and 30 °C, respectively. The samples were eluted at a flow rate of 0.3 mL/min using two mobile phases (A: 0.1% formic acid in ultrapure water; B: 0.1% formic acid in 50:50 ultrapure water:acetonitrile) with the following gradient: 0 min 5% B; 4 min 5% B; 32.00 min 100% B; 34.00 min 100% B; 36.00 min 5% B; 40.00 min 5% B.

For optimal electrospray ionization conditions, the nebulizer pressure, drying gas temperature, and drying gas were set to 45 psi, 350 °C, and 3 L/min, respectively. In addition, the DAD was performed at 254, 280, and 520 nm. Morin levels (internal standard) were integrated in Agilent Mass Hunter Quan software (v. B.06.00, Cheadle, UK), and all samples with deviations larger than 10% relative to the dataset mean were reinjected. For all samples, three aliquots were analyzed across three different analytical batches.

### 2.4. Component Detection, Peak Alignment, and Integration

All chromatograms were evaluated in the same manner using the Agilent Software Profinder v. B.06.00, as previously described by Dudnik et al. [12], which integrates peak findings in an automated and unbiased way with a peak integration user interface that allows user-driven curation of individual peaks. For positive mode data, the batch recursive molecular feature was used with peak extraction restricted to 2.1–38.00 min of the chromatography and peaks with levels higher than 15,000 counts with potential adducts of +H, +Na^+^, +K^+^, and +NH_4_^+^ (−H and +Cl^−^ in negative mode) and a maximum of one charge state. The compound ion count threshold was set at two or more ions, and for alignment purposes the RT window was set at 0.70% + 0.60 min and the mass window was set at 25 ppm + 2 mDa. A post-processing filter to restrict analysis to compounds with more than 15,000 counts and present in at least 3 of the files in at least one sample group (species/line). The find-by-ion options were set to limit the extracted ion chromatogram (EIC) to the expected retention time +/− 0.40 min. The “Agile” algorithm was used for the integration of EIC, with a Gaussian smoothing of 9 points applied before the integration and a Gaussian width of 3 points. Additionally, peak filters were set at over 15,000 counts and the chromatogram formats were set to centroid when available and otherwise profile. The spectrum was extracted at 10% of peak height and excluded if the spectra within the *m*/*z* range used was above 20% of the saturation. Finally, a post-processing filter was applied and compounds with less than 15,000 counts or present in less than 3 files in at least one sample group (species/line) were excluded. The output of automated peak finding and integration resulted in 542 and 210 molecular features found in the positive and negative modes, respectively. Manual curation resulted in the narrowing down of the molecular features found to 366 and 169 in the positive and negative modes, respectively. These were subsequently used in the statistical analysis.

### 2.5. Multivariate Analysis

GenStat for Windows, 16th Edition (VSN international Ltd., Hemel Hempstead, UK) was used for all the multivariate analysis performed. A principal component analysis (PCA), based on the correlation matrix, was applied to all the QC samples to ensure that the blank, reference samples, and berry samples were well separated (data not shown). The positive and negative metabolite datasets were analyzed separately and PCA plots were generated for the first 4 principal components. These were subsequently used for selecting the species with the greatest phytochemical differences.

### 2.6. Yeast Strains, Plasmids, and Transformation

Strains and plasmids used in this study are listed in Tables S3 and S4, respectively. The W303-1A_FUS and W303-1A_T strains were obtained by transformation of the W303-1A strain with plasmids pAG303_GAL1pr-FUS and pAG303_ GAL1pr-ccdB previously linearized with *BstZ*17I. Yeast transformation procedures were carried out as indicated using the lithium acetate standard method [28].

### 2.7. Yeast Growth Conditions

Synthetic complete (SC) medium (0.67% yeast nitrogen base without amino acids (YNB) (Difco^TM^ Thermo Scientific Inc., Waltham, MA, USA) and 0.79 g/L complete supplement mixture (CSM) (MP Biomedicals, Inc.—Fisher Scientific, Irvine, CA, USA)), containing 1% raffinose was used for growth of PD and ALS integrative yeast models. Synthetic dropout CSM_-URA_ medium (0.67% YNB and 0.77 g/L single amino acid dropout CSM_−URA_ (MP Biomedicals, Inc.—Fisher Scientific, Irvine, CA, USA)) containing 1% raffinose was used for growth of AD and ALS episomal yeast models. For growth of the HD model, a synthetic dropout SC-LEU medium was used (0.67 % YNB and 0.54 g/L 6-amino acid dropout CSM_–ADE–HIS–LEU–LYS–TRP–URA_ (MP Biomedicals, Inc.—Fisher Scientific, Irvine, CA, USA), supplemented with standard concentrations of the required amino acids and containing 1% (*w/v*) raffinose. For growth of the RAS–RAF interaction yeast model, CSM_–HIS–URA–TRP_ media was used (0.67% YNB and 0.54 g/L 6-amino acid dropout CSM_–ADE–HIS–LEU–LYS–TRP–URA_ (MP Biomedicals, Inc.—Fisher Scientific, Irvine, CA, USA), supplemented with standard concentrations of the required amino acids and containing 1% raffinose. In all conditions, medium containing glucose (control, disease-protein OFF) and galactose (disease-protein ON), at a final concentration of 2%, were used for the repression or induction of disease protein expression, respectively. Growth of Crz1 activation yeast model was performed in SC medium containing 2% (*w/v*) glucose, and Crz1 activation was induced with 1.8 mM MnCl_2_ [29]. Radicicol (Sigma, Gillingham, UK) and FK506 (Cayman Chemicals, Ann Arbor, MI, USA) were used as positive controls for the yeast models of RAS–RAF interaction and Crz1 activation, respectively.

A pre-inoculum was prepared in raffinose or glucose (for Crz1 activation model) medium. Cultures were incubated overnight at 30 °C under orbital shaking, diluted in fresh medium, and incubated under the same conditions until the optical density at 600 nm (OD_600_) reached 0.5 ± 0.05 (log growth phase). Cultures were then diluted according to the equation: ODi × Vi = (ODf/(2^(*t*/*gt*)^) × *Vf*, where ODi = initial optical density of the culture, Vi = initial volume of culture, ODf = final optical density of the culture, *t* = time (usually 16 h), *gt* = generation time of the strain, and *Vf* = final volume of culture. Readings were performed in a 96 well microtiter plate using a Biotek Power Wave XS plate spectrophotometer (Biotek^®^ Instruments, Winooski, VT, USA). Dried extracts of *Rubus* species/cultivar obtained after total phenolic compounds determination were re-solubilized in adequate growth medium for the cellular assays.

### 2.8. Growth Assays

For the phenotypic growth assays, the strains were grown as described to OD_600_ 0.1 ± 0.01 and were inoculated (OD_600_ 0.2 ± 0.02) in medium supplemented or not with the indicated concentrations of *Rubus* extracts. After 6 h, OD_600 nm_ was adjusted to 0.05 ± 0.005, serial dilutions were performed with a ratio of 1:3, and 5 μL of each dilution was spotted onto solid medium containing glucose or galactose as the sole carbon sources. Growth was recorded after 48 h incubation at 30 °C. Images were acquired using Chemidoc_TM_ XRS and Image-Lab^®^ 6.0.1 software (Biorad, Hercules, CA, USA). For the growth curves, yeast cultures were diluted to OD_600_ 0.12 ± 0.012 in fresh medium supplemented or not with the indicated concentrations of the extracts in a 96 well microtiter plate. After 2 h incubation at 30 °C, cultures were further diluted to OD_600_ 0.03 ± 0.003 in repressing (glucose) or inducing (galactose) media supplemented or not with the extracts. The cultures were incubated at 30 °C with shaking for 24 h or 48 h (for the AD model) and cellular growth was monitored hourly by measuring OD_600_ using a Biotek Power Wave XS Microplate Spectrophotometer (Biotek^®^ Instruments, Winooski, VT, USA). The areas under the curve (AUC) were integrated using the Origin 6 software (OriginLab, Northampton, MA, USA). For the RAS/RAF interaction model, final biomass was calculated by normalizing OD_600_ of cultures after 48 h incubation at 30 °C to the initial OD_600_.

### 2.9. Flow Cytometry

Cell cultures at OD_600_ 0.2 ± 0.02 were exposed or not to the indicated concentrations of *Rubus* extracts for 6 h. Cultures were further diluted to OD_600_ 0.2 ± 0.02 in glucose and galactose supplemented or not with the indicated concentrations of *Rubus* extracts for 12 h. Flow cytometry was performed in a FACS BD Calibur equipped with a blue solid-state laser (488 nm) and green fluorescence channel 530/30 nm. Data analysis was performed using FlowJo software (BD, San Jose, CA, USA), and the cell doublets exclusion was performed based on Forward-A and -W scatter parameters. A minimum of 30,000 events were analyzed for each experiment. Results are expressed as the percentage of GFP positive cells as compared to the control.

### 2.10. Fluorescence Microscopy

Yeast cells subjected to the same treatment as above were monitored for the formation of disease-protein intracellular inclusions or nuclear translocation of Crz1 by fluorescence microscopy using a Leica DMRA2 fluorescence microscope (Leica, Wetzlar, Germany) equipped with a CoolSNAP HQ CCD camera (1.3MPx monochrome). Images were analyzed using ImageJ 1.8.0 software (NIH, Bethesda, MD, USA).

### 2.11. Protein Extraction

Tris-based buffer (TBS) [30] or trichloroacetic acid (TCA)/MURB (50 mM sodium phosphate, 25 mM MES pH 7.0, 1% SDS, 3 M urea, 0.5% 2-mercaptoethanol, 1 mM sodium azide) [31] were used for total protein extraction. Aliquots corresponding to OD_600_ 1–2 of cultured cells were harvested by centrifugation at 5000× *g* for 3 min. For TBS extraction, cells were resuspended in TBS supplemented with protease and phosphatase inhibitors, disrupted with glass beads (3 cycles of 30 s in the vortex and 5 min on ice), and cell debris were removed by centrifugation at 700× *g* for 3 min. Total protein was quantified using the MicroBCA kit (Thermo Fisher Scientific, Waltham, MA, USA) according the manufacturer’s instructions. Samples were incubated at 95 °C for 10 min before SDS-PAGE.

For the TCA/MURB protocol, cells were first resuspended in TCA to a 10% final concentration, and the samples were incubated for 20 min at −20 °C. The cells were harvested by centrifugation at 15,000× *g* for 3 min, washed twice with acetone, and the air-dried cell pellet was resuspended in MURB supplemented with protease and phosphatase inhibitor cocktails. Cells suspensions were disrupted with glass beads (3 cycles of 30 s in the vortex and 5 min on ice), the samples were incubated at 70 °C for 10 min, and unlysed cells were removed by centrifugation at 10,000× *g* for 1 min.

### 2.12. Immunoblotting

Equal volumes of protein extract, normalized to the OD_600_ of cell cultures (for TCA/MURB protocol), or equal concentrations of total proteins (for TBS protocol) were loaded in a 15% SDS-PAGE. The Trans-Blot Turbo transfer system (BioRad, Hercules, CA, USA) was used to transfer proteins to a 0.22 µm nitrocellulose membrane according to the manufacturer’s specifications. Membranes were washed with TBS, blocked with 5% skim milk in TBS-Tween for 1 h at room temperature, and incubated overnight at 4 °C with antibodies against GFP (1:5000, Neuromab, Davis, CA, USA), FUS/TLS (1:1000, Millipore, Burlington, MA, USA), and PgK1 (1:5000, Life Technologies Corporation, Carlsbad, CA, USA). Membranes were washed three times with TBS-Tween and incubated with horseradish peroxidase-conjugated secondary antibodies (1:10,000, Pierce, Waltham, MA, USA) for 2 h at room temperature. Protein signals were detected using Amersham ECL Prime Detection Reagent (GE Healthcare, Chicago, IL, USA) and signal intensity was estimated using the ImageJ 1.8.0 software (NIH, Bethesda, MD, USA).

### 2.13. β–Galactosidase Assays

For monitoring of RAS/RAF interaction, cell cultures at OD_600_ 1 ± 0.1 were exposed or not to the indicated concentrations of *Rubus* extracts for 90 min. The extracts were removed, cells were patched at a density of 4.5 × 10^7^ onto solid medium containing glucose or galactose, and incubated 3 h at 30 °C. The assay was revealed by overlaying the cells with 5-Bromo-4-Chloro-3-Indolyl β-d-Galactopyranoside (X-Gal) solution (0.5% agarose, 50% LacZ buffer, 0.2% SDS, 2 mg X-Gal/mL and at 70 °C). Plates were maintained at 30 °C and monitored until the development of the blue color [32].

For quantitative measurements of β–galactosidase activity, cell cultures at OD_600_ 0.5 ± 0.05 were diluted to OD_600_ 0.1 ± 0.01 and challenged or not with extracts and the pure compounds for 90 min. Just before cell lysis, OD_600_ of cultures were recorded. Cells were incubated with Y-PER Yeast Protein Extraction Reagent (ThermoFisher Scientific) in 96 well microtiter plates for 20 min at 37 °C, LacZ buffer containing 4 mg/L 2-Nitrophenyl β-d-galactopyranoside (ONPG) was added, and plates were incubated at 30 °C [32]. The OD_420_ and OD_550_ were monitored using a Biotek Power Wave XS Microplate Spectrophotometer (Biotek^®^ Instruments, Winooski, VT, USA). Miller units were calculated as described previously [33].

### 2.14. Quantitative Real-Time PCR

The qRT-PCR analyses were performed according to the MIQE guidelines (Minimum Information for Publication of Quantitative Real-Time PCR Experiments) [34]. Total RNA was extracted using the ENZA yeast RNA extraction kit (OMEGA, Norcross, GA, USA). After cleaning, 200–300 ng of total RNA was used for reverse-transcription with qScript™ cDNA superMix kit (Quanta Biosciences Inc., Gaithersburg, MD, USA). The qRT-PCR was performed in a LightCycler 480 Instrument (Roche, Basel, Switzerland), using LightCycler 480 SYBR Green I Master (Roche, Basel, Switzerland) to evaluate expression of the *FUS–GFP* (5′-ACGGACACTTCAGGCTATGG-3′; 5′-CCCGTAAGACGATTGGGAGC-3′) (GeneID: 2521), *PMR1* (5′-CACCTTGGTTCCTGGTGATT-3′; 5′-CCGGTTCATTTTCACCAGTT-3′) (GeneID: 852709), *PMC1* (5′-GTGGCGCACCATTTTCTATT-3′; 5′-TACTTCATCGGGGCAGATTC-3′) (GeneID: 852878), and *GSC2* (5′-CCCGTACTTTGGCACAGATT-3′; 5′-GACCCTTTTGTGCTTTGGAA-3′) (GeneID: 852920) genes. Standard curves were constructed for each gene and expression was calculated by the relative quantification method with efficiency correction using LightCycler 480 Software version 1.5.0.39 (Roche, Basel, Switzerland). Both *ACT1* (5′-GATCATTGCTCCTCCAGAA-3′; 5′-ACTTGTGGTGAACGATAGAT-3′) and *PDA1* (5′-TGACGAACAAGTTGAATTAGC-3′; 5′-TCTTAGGGTTGGAGTTTCTG-3′) were used as reference genes. The results were expressed as fold-change mRNA levels relative to the control (mRNA fold change) of at least three independent biological replicates.

### 2.15. Microglia-Induced Inflammation Model

Microglial N9 cells were cultured in EMEM (Eagle Minimum Essential Media, Sigma–Aldrich, Gillingham, UK) media, supplemented with 1% (*v*/*v*) *L*-glutamine (Biochrom AG, Berlin, Germany), 1% (*v*/*v*) penicillin/streptomycin, and 10% FBS (Fetal Bovine Serum, Gibco, Waltham, MA, USA). Cell cultures were maintained at 37 °C in 5% (*v*/*v*) CO_2_, and split at sub-confluent cultures (about 60–80%). Cells were then detached by agitation before suspension of the culture media with a pipette (no cellular detaching agent was used). For immunostaining, cells were grown at 5 × 10^4^ cell/well in 24 well plates containing coated coverslips and cultured overnight. Cells were pre-incubated or not with 5 mM of the indicated compounds for 6 h in culture media with reduced FBS to 0.5% (*v*/*v*). The medium was discarded, and cells were washed with PBS. Fresh culture media containing 300 ng/mL of LPS (Lipopolysaccharide) or 3 mM ATP (Sigma-Aldrich–Poole, Gillingham, UK) was added and cell cultures were incubated for 1 h to induce transcription factor nuclear localization. For nitric oxide (NO) and tumor necrosis factor alpha (TNF-α) quantifications, cells were seeded at 5 × 10^5^ cells/well in 6 well plates, cultured overnight, and pre-incubated or not with 5 mM of the indicated compounds for 6 h in culture media with reduced FBS to 0.5% (*v*/*v*). The medium was discarded, cells were washed with PBS, and incubated in fresh culture media containing 300 ng/mL of LPS for 24 h.

### 2.16. Immunofluorescence

Immunostaining was performed as described by Figueira et al. [35], using rabbit polyclonal anti-NF-κB p65 (C-20) (1:200, Santa Cruz Biotechnology, Santa Cruz, CA, USA) or rabbit polyclonal anti-NFAT1c (1:200, Cell Signalling, Danvers, MA, USA) as primary antibodies and Alexa 594 anti-rabbit IgG (1:500) (Invitrogen, Carlsbad, CA, USA) as the secondary antibody. Nuclei were counterstained with DAPI. Cells were washed three times with PBS between each incubation. Widefield images were acquired on a Leica DMRA2 upright microscope, equipped with a CoolSNAP HQ CCD camera, using a 63× 1.4NA oil immersion objective, DAPI + TRITC fluorescence filter sets. Post-acquiring treatment was performed using ImageJ 1.8.0 software (NIH, Bethesda, MD, USA).

### 2.17. Nitric Oxide (NO) Quantification

The NO release to media was quantified as described by Ii et al. [36] using the Griess Reagent (Sigma–Aldrich, Gillingham, UK), according to manufacturer’s instructions. After incubation with LPS, cell media were removed and immediately analyzed for nitrite quantification. Standard curves of sodium nitrite (0–25 μmol/L) were prepared and absorbance was acquired in a Synergy HT microplate reader (Biotek^®^ Instruments, Winooski, VT, USA).

### 2.18. TNF-α Quantification

The TNF-α release was assayed by ELISA according to the manufacturer’s instructions (PeproTech^®^; Princeton Business Park, Rocky Hill, NJ, USA). All reagents and plates used were provided in the kit. For the standard curve, recombinant murine TNF-α (PeproTech^®^) was diluted from 0–2 μg/L. The plate was incubated at room temperature in a Synergy HT microplate reader (Biotek^®^ Instruments, Winooski, VT, USA) for 35  min, with 5 min intervals Abs_405_ readings.

### 2.19. Statistical Analysis

The results reported in this study are the average of at least independent biological triplicates and are represented as the mean ± SEM. Analysis of variance with Tukey’s HSD (Honest Significant Difference) multiple comparison test (α = 0.05) using SigmaStat 3.10 (Systat, Chicago, IL, US) was used to assess the differences among treatments.

## 3. Results

### 3.1. Selection of Most Chemically Diverse Species

**Figure 1:**
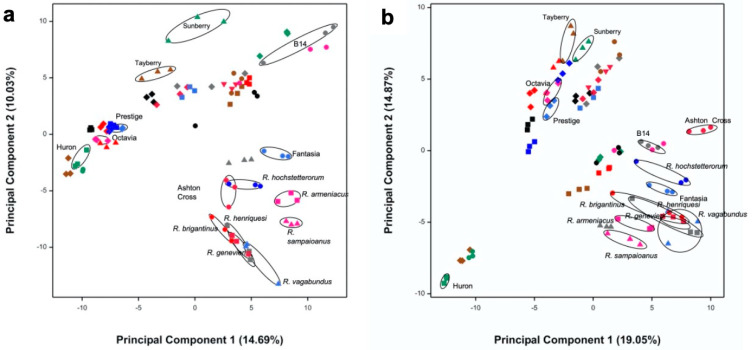
Principal component analysis (PCA) plots of the first two principal components (PCs) for the untargeted analysis acquired in positive (**a**) and negative (**b**) modes. These plots illustrate the phytochemical diversity present within the *Rubus* germplasm collection analyzed which included 36 species/cultivars. In positive mode, it was possible to identify three major groups comprising black and red raspberries (negative values for principal component 1, PC1), hybrid berries (high positive values for principal component 2, PC2), and wild and some domesticated blackberries (positive for PC1 and negative for PC2). In negative mode, three main groups were also observed, although they comprised black raspberries (negative for PC1 and PC2), red raspberries and hybrid species (high positive values for PC2), and wild and domesticated blackberries clustering with some hybrid species (positive values for PC1). The species selected for evaluation of bioactivity are circled in both plots.

(Poly)phenol-enriched extracts were prepared from a *Rubus* germplasm collection composed of 36 species/cultivars (Table S1). These were analyzed by LC-ToF-MS with the goal of selecting the most phytochemically diverse species. This was achieved using an untargeted analysis of extracts in both positive and negative modes which produced a total of 535 distinct molecular features for the entire dataset and subsequently combined with multivariate statistical analysis. There was no significant effect caused by instrument variability (see Figures S1 and S2), and principal component analysis (PCA) on the correlation matrix of the *Rubus* molecular features showed groupings of samples, with the majority of samples clustered according to their species/cultivar, reflecting their phytochemical similarity (Figure 1).

We selected for further bioactivity assessment the species/samples showing the largest separation on the PCA plots (the first four principal components) generated from the positive and negative modes, as they were likely to represent the highest variability in phytochemical composition. Therefore, a total of 15 cultivars/species extracts were selected for screening in the SMART discovery platform: *Rubus vagabundus* Samp. *Rubus brigantinus* Samp., *Rubus sampaioanus* Sudre ex Samp., *Rubus genevieri* Boreau., *Rubus hochstetterorum* Seub, *Rubus henriquesii* Samp., *Rubus loganobaccus* L.H. Bailey (var. Tayberry and var. Sunberry), *Rubus fruticosus* L. *agg* (var. Fantasia, var. Ashton cross), *Rubus idaeus* L. (var. Prestige, var. Octavia), *Rubus occidentalis* L. (var. Huron), *Rubus armeniacus* Focke (var. Himalayan giant), and *Rubus* spp. (James Hutton Institute accession number B14).

### 3.2. Identification of Bioactivities Using a SMART Discovery Platform

Our approach involved the exploitation of a yeast-based screening platform, which was used to assess the potential of bioactives to modulate specific pathological pathways associated with redox-related diseases, particularly, neurodegenerative diseases, cancer, and inflammation (Figures S3–S8).

The most chemically diverse samples were screened in this discovery platform, and the protection factor for each pathological process was determined (Table 1). Protection factors above 10% were considered positive. The protection assays were preceded by cytotoxicity assays using control strains to determine the maximum useable extract concentration that caused less than 20% toxicity. Only the *R. idaeus* (var. Octavia) extract slightly improved the growth of αSyn-expressing cells (Parkinson’s disease-PD-model), whereas *R. loganocaccus* (var. Sunberry) efficiently rescued the growth of yeast cells expressing Aβ (Alzheimer’s disease-AD-model).

Several bioactivities for cellular pathologies associated with Huntington’s disease (HD) and amyotrophic lateral sclerosis (ALS) were identified. *R. idaeus* (var. Prestige), and *R. loganobaccus* (var. Sunberry) extracts almost restored the growth of cells expressing mutant HTT (HD model) to values comparable to those of control cells. In cells expressing FUS (ALS model), the Portuguese endemic species *R. genevieri and R. idaeus* (var. Prestige) yielded the most potent extracts. *R. occidentalis* (var. Huron) conferred similar levels of protection (~57%) in KRAS/BRAF and Crz1–*lacZ* models. Remarkably, inflammation was the pathological process with a higher number of positive hits, as 10 out of 15 extracts tested conferred protection in the Crz1–*lacZ* model.

As the most potent extract among the Portuguese endemic species against FUS-mediated toxicity and conferring the highest level of protection in the KRAS/BRAF model, *R. genevieri* and *R. occidentalis* (var. Huron) extracts were chosen to obtain further insight into their mode of action towards ALS and cancer, respectively. Because inflammation is a central pathological process of neurodegenerative diseases, such as HD, and *R. idaeus* (var. Prestige) was previously shown by us to confer protective activities against this disease [13], we also included the characterization of the anti-inflammatory potential of this extract in the present study.

### 3.3. Bioactivity Towards the Mitigation of FUS Proteotoxicity

**Figure 2:**
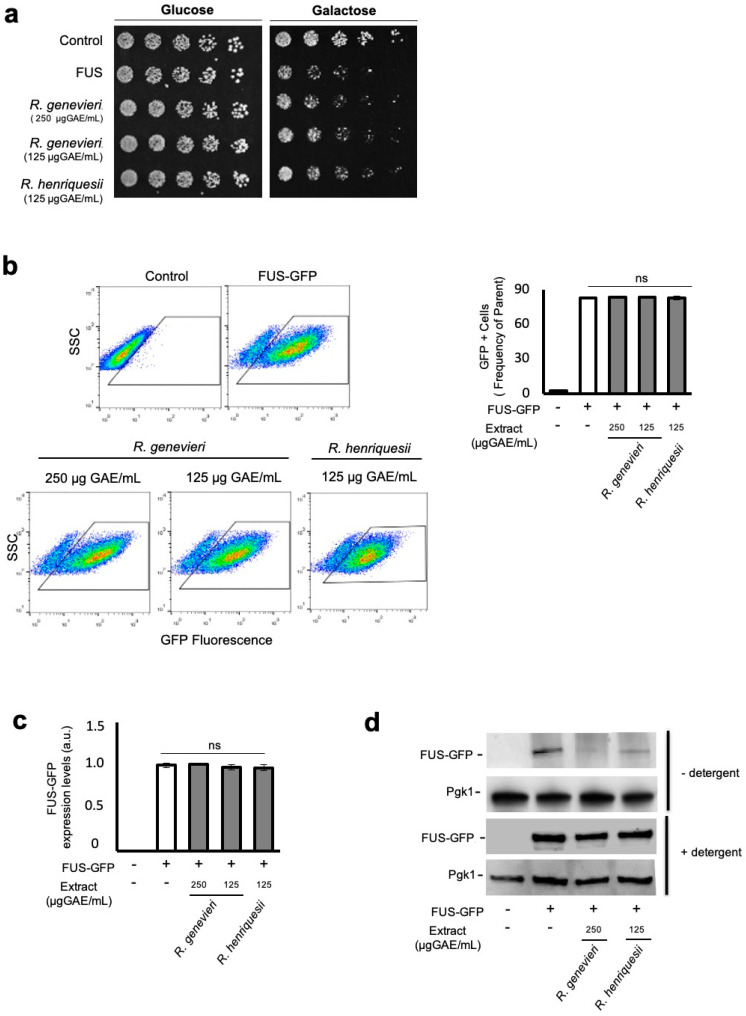
Bioactivity towards Amyotrophic lateral sclerosis is associated with the reduction of soluble FUS levels by *R. genevieri*. W303-1A recombinant cells expressing FUS were pre-grown in synthetic complete raffinose medium, and cells encoding the empty vector were used as the control. (**a**) The viability of cells exposed or not to the indicated concentrations of phenolic compound-enriched extracts was assessed by phenotypic growth assays on synthetic complete glucose and synthetic complete galactose media. (**b**) FUS expression evaluated by side scatter (SSC) versus FUS–GFP fluorescence, assessed by flow cytometry (left panel). The percentage of FUS–GFP-positive cells is shown (right panel). (**c**) FUS–GFP expression levels as evaluated by qRT-PCR. (**d**) FUS–GFP protein levels upon extraction in the presence or absence of detergent as evaluated by immunoblotting. Representative images are shown, and the values represent the mean ± SEM of at least three biological replicates.

*Rubus genevieri* extracts conferred significant protection for the FUS-expressing model at the final concentration of 250 µg GAE/mL (Table 1), the higher non-toxic concentration tolerated by cells as defined in the cytotoxicity assays (data not shown). We then performed phenotypic growth assays to evaluate extract bioactivity as a pre-treatment. This condition contrasts with those used in the discovery platform, where cells were exposed to the extracts simultaneously with the induction of FUS expression. Cells expressing FUS displayed reduced growth in comparison with the control strain (Figure 2a and Figure S4b,c). Pre-treatment with *R. genevieri* (poly)phenol-enriched extracts partially rescued cellular growth, consistent with the screening assays, even when half the concentration (125 µg GAE/mL) was used (Figure 2a). Extracts from *R. henriquesii* slightly protected cells from FUS toxicity when applied as a pre-treatment (Figure 2a) in contrast to the screening assays where no protection was detected. Growth improvement mediated by *R. genevieri* phenolics was neither associated with alterations in the percentage of GFP positive cells (Figure 2b) nor FUS–GFP mRNA levels (Figure 2c). Analysis of protein levels by immunoblotting indicated that treatment with *R. genevieri* phenolics decreased FUS–GFP levels as compared to the control condition (Figure 2d, upper panels). As flow cytometry showed no variation of GFP signals among conditions, we assessed whether treatment with *R. genevieri* phenolics affected FUS biochemical status using a detergent solubility assay. In agreement with the flow cytometry results, no differences in FUS–GFP levels were observed under these conditions (Figure 2d, lower panels). These data suggest that *R. genevieri* phenolics may induce the formation of insoluble intracellular FUS structures.

### 3.4. Bioactives Modulating RAS/RAF Pathological Interactions

**Figure 3:**
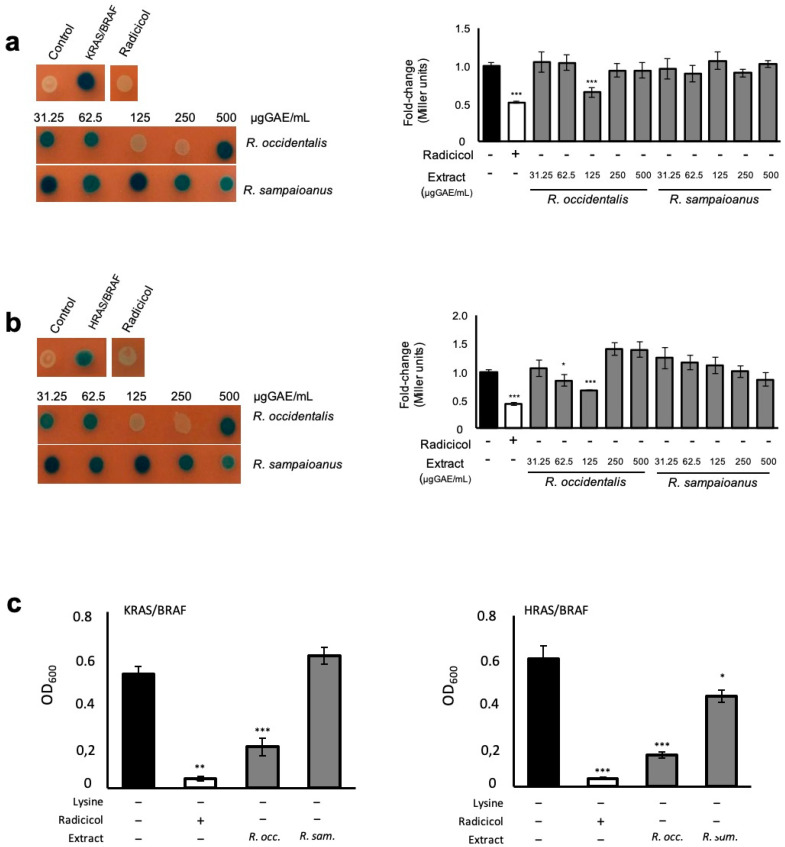
Mitigation of the pathological interaction between RAS and RAF by *R. occidentalis* (var. Huron). SKY197 recombinant cells expressing *ADH1pr-*λCI-RAS *GAL1pr-B42-BRAF* from 2µ vectors were pre-grown in synthetic dropout raffinose medium and exposed or not to the indicated concentrations of phenolic compounds-enriched extracts. Radicicol was used as a positive control. Cells containing the empty vectors were used as control. KRAS/BRAF (**a**) and HRAS/BRAF (**b**) interaction was assessed by monitoring β-galactosidase activity in SD galactose medium using 5-Bromo-4-Chloro-3-Indolyl β-d-Galactopyranoside (X-Gal) (left panels) and 2-Nitrophenyl β-d-galactopyranoside (ONPG) (right panels). (**c**) RAS/RAF interaction assessed by monitoring the final biomass of cell cultures grown in SD galactose medium without lysine and supplemented with 125 µg GAE/mL of the indicated phenolic compounds-enriched extracts. Representative images are shown, and the values represent the mean ± SEM of at least three biological replicates, * *p* < 0.05, ** *p* < 0.01, *** *p* < 0.001. *R. occ.*—*R. occidentalis*; *R. sam.*—*R. sampaioanus*.

*Rubus occidentalis* (var. Huron) was the most potent in reducing the KRAS/BRAF interaction (Table 1). We performed a dose–response analysis to determine the concentration range of *R. occidentalis* (var. Huron) phenolics that conferred protective activity. Radicicol and *R. sampaioanus* extract (Table 1) were used as positive [37,38] and negative controls, respectively. Radicicol inhibited KRAS/BRAF interaction to levels comparable to the control strain bearing the empty plasmids (Figure 3a, left panel). Extracts from *R. occidentalis* (var. Huron) prevented KRAS/BRAF interaction at a concentration range of 125–250 µg GAE/mL, whereas *R. sampaioanus* had no significant effect on the interactions among these proteins. Similar results were obtained when β-galactosidade activity was measured in liquid medium (Figure 3a, right panel), except that only the lower concentration of *R. occidentalis* exhibited inhibitory activity on KRAS/BRAF interactions. Evaluation of BRAF interaction with the HRAS isoform revealed that *R. occidentalis* (var. Huron) bioactivity was maintained (Figure 3b), further supporting the protective role of (poly)phenolics present in this extract towards pathological RAS/RAF interactions. Notably, *R. occidentalis* (var. Huron) blocked RAS/RAF to a similar level to that of radicicol. No effects were observed in cells where the expression of BRAF-B42 fusion was turned off (Figure S9a,b).

The RAS/BRAF interaction was also assessed using *LYS2* as a reporter (Figure S7e). For that, cells co-expressing the control constructs, KRAS/BRAF and HRAS/BRAF were treated with the indicated extracts and the final biomass of cultures were monitored after 48 h incubation at 30 ºC. As depicted in Figure 3c, the *R. occidentalis* (var. Huron) extracts mediated a strong protection towards BRAF interaction with both RAS isoforms, whereas *R. sampaioanus* had only a marginal effect on HRAS/BRAF interaction. Modulation of RAS/RAF interaction by *R. occidentalis* (var. Huron) (poly)phenolics occurred under subtoxic concentrations when the final biomass of cultures grown in galactose or glucose medium supplemented with lysine was measured (Figure S9c). Similar results were observed for the control cells expressing the empty plasmids (Figure S9d).

### 3.5. Attenuation of Crz1 Activation 

**Figure 4:**
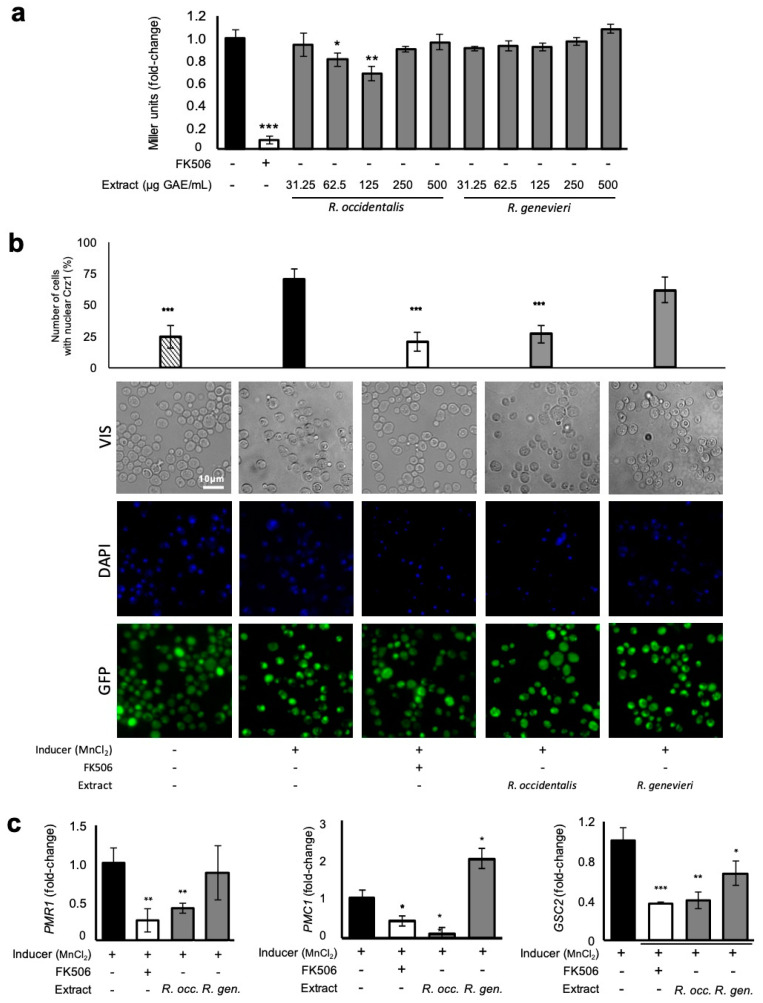
Attenuation Crz1 activation by *Rubus occidentalis* (var. Huron). BY4741 yeast strains encoding *CRZ1^promoter^–lacZ* or *CRZ1–GFP* were subjected or not to the indicated concentrations of phenolic compounds-enriched extracts and induced with 1.8 mM MnCl_2_. (**a**) Crz1 activation was assessed by monitoring β-galactosidase activity using 2-Nitrophenyl β-d-galactopyranoside (ONPG). (**b**) Crz1 subcellular dynamics evaluated by fluorescence microscopy in cells treated with 125 µg GAE/mL of the indicated phenolic compounds-enriched extracts. (**c**) Activation of Crz1 target genes *PMR1*, *PMC1*, and *GSC2* in cells treated as above by means of qRT-PCR. The immunosuppressant FK506, which inhibits calcineurin and prevents Crz1 activation, was used as a positive control. Representative images are shown, and the values represent the mean ± SEM of at least three biological replicates, * *p* < 0.05, ** *p* < 0.01, *** *p* < 0.001.

(Poly)phenolic-enriched extracts were also tested for their potential to reduce Crz1 activation (Figure S8). *Rubus occidentalis* (var. Huron) extract was one of the most potent at inhibiting Crz1 activation (Table 1). The dose–response analysis indicated that this protection was restricted to the concentration range of 62.5–125 µg GAE/mL. Lower (31.25 µg GAE/mL) and higher (250 µg GAE/mL) concentrations had no effect on Crz1 activation and neither did the extract from *R. genevieri* at all concentrations tested (31.25–500 µg GAE/mL) (Figure 4a). These results emphasize the specificity of *R. occidentalis* (var. Huron) (poly)phenolics. The immunosuppressant FK506, which inhibits calcineurin activation thereby preventing Crz1 activation, was used as a positive control. In contrast to FK506, which was shown to reduce Crz1 basal activity, *R. occidentalis* (var. Huron) protection is specific for conditions in which cells were exposed to MnCl_2_, a well-known inducer of Ca^2+^-signaling pathway and Crz1 activity (Figure S10).

In a conserved mechanism to NFAT, Crz1 is dispersed throughout the cell under non-inducing conditions. Upon external stimuli leading to the increase of Ca^2+^ cytosolic levels, dephosphorylated Crz1 (and NFAT) accumulates in the nucleus where it binds to calcineurin-dependent response element (CDRE) and activates transcription of the target genes. The Crz1–GFP construct was used to further evaluate the potential of *R. occidentalis* (var. Huron) (poly)phenolics to modulate Crz1 activation by controlling its subcellular localization. As expected, GFP fluorescence signals were observed throughout the cell under physiological conditions. Exposure to MnCl_2_, a well-known inducer of Ca^2+^-signaling pathway and Crz1 activity, led to the rapid translocation of Crz1 into the nucleus (Figure 4b and Figure S8d), which was partially abolished by treatment with FK506 as indicated by the reduced number of cells displaying nuclear GFP signals (Figure 4b). *Rubus occidentalis* (var. Huron), but not *R. genevieri* extracts, reduced the percentage of cells with nuclear GFP indicating that this extract controls Crz1 activity by preventing its accumulation in the nucleus. If (poly)phenolics from *R. occidentalis* (var. Huron) prevent Crz1 nuclear translocation, it can be hypothesized that activation of Crz1 regulon should also be affected. Thus, we monitored by qPCR the mRNA levels of *PMR1*, *PMC1*, and *GSC2* Crz1-regulated genes. Fully supporting our hypothesis, the treatment of cells with *R. occidentalis* (var. Huron) extract downregulated expression of the three genes to a similar extent of that of FK506, whereas *R. genevieri* extracts had only a minor effect on the expression of *GSC2* (Figure 4c).

### 3.6. Runs Test for the Selection of Potential Components with Bioactivities

The qualitative and quantitative diversity present in the extracts ultimately translated into differences in the specificity and efficacy of the extracts in modulating bioactivity in the described disease models. Although an association between phytochemical composition and bioactivity is apparent, few studies have studied the association between bioactivities and the phytochemical composition of phylogenetically related species [39]. While correlation analysis approaches using metabolite and activity levels have been previously used in models of cytotoxicity or enzymatic activity where a dose-response is often observed between metabolite and bioactivity, it may result in false negative errors in the analysis of cell-based bioactivity models, which may experience additional cytotoxic effects at high metabolite concentrations. Similarly, a test of means of metabolite concentrations in bioactive extracts versus non-bioactive extracts may result in false negative results when there is a toxicity response at higher metabolite concentrations. To mitigate this, for each molecular feature the species were ordered according to their levels and a runs test was used on the bioactivity levels (+/−) of this order to test the randomness of the conditional distribution (molecular feature levels) given the observations of bioactivity (+) or lack of bioactivity (−) (Figure S11). This detects any significantly larger than expected number of runs of the same level of bioactivity as molecular feature levels increase. Out of the 535 molecular features in the metabolite dataset, the runs test yielded 48 molecular features with potential effects on whether a berry extract is active or inactive (*p* < 0.05). These results were further filtered in order to match two expected models of response for bioactivity: (a) higher molecular feature levels associated with bioactive extracts or (b) higher levels of molecular features associated to bioactivity and a lack of bioactivity observed at the highest levels due to the cytotoxic effects (Figure S11). This allowed the reduction of 48 potential molecular features to 15 tentatively annotated metabolites with potential bioactivities for four disease models (Table S2). This analysis provided three potential bioactives for HTT toxicity, nine hits for KRAS/RAF interaction, one hit for FUS toxicity, and two hits for Crz1 activation (Table S2). As there is only one extract each with reported bioactivity for Aβ42 and αSyn toxicity (Table 1), the statistical analysis returned no significant compounds (*p* < 0.05).

### 3.7. Unveiling Cyanidin as the Anti-Inflammatory Molecule

**Figure 5:**
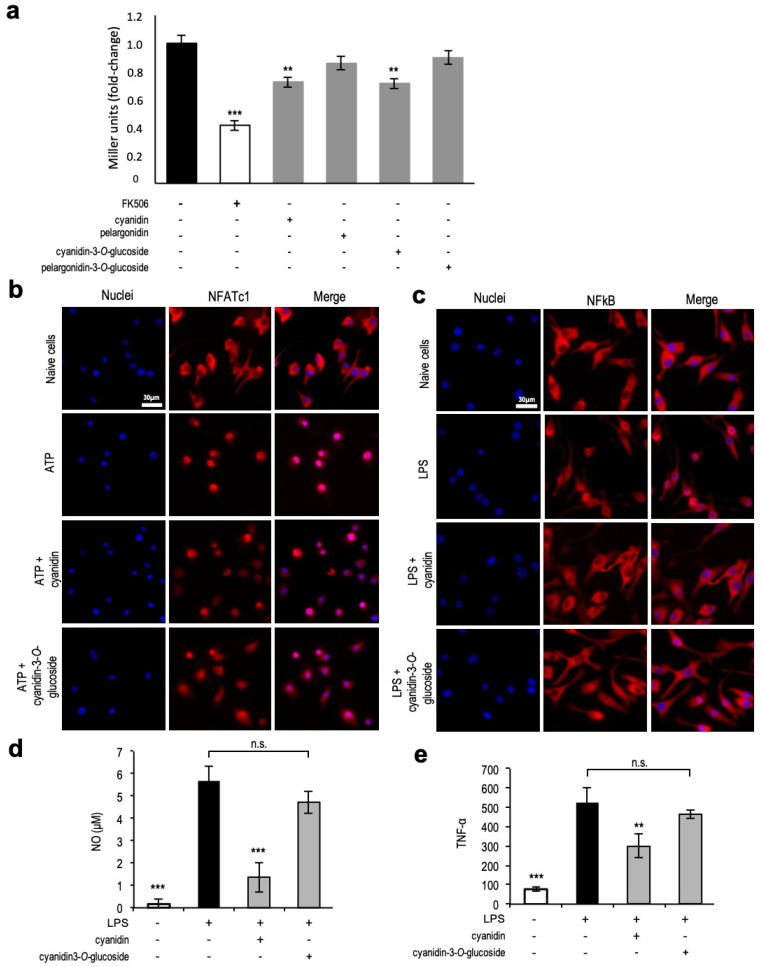
Cyanidin modulates Crz1 activation in yeast and NFAT and NF-kB in microglia. (**a**) BY4741 yeast strain encoding *CRZ1^promoter^–lacZ* was subjected or not to 50 µM of the indicated compounds, induced with 1.8 mM MnCl_2_, and Crz1 activation was assessed by monitoring β-galactosidase activity using 2-Nitrophenyl β-d-galactopyranoside (ONPG). The immunosuppressant FK506 was used as a positive control. (**b**) NFATc1 and (**c**) NF-kB translocation to the nucleus in N9 cells pre-treated with 5 µM cyanidin or cyanidin-3-*O*-glucoside for 6 h before 1 h ATP stimulation (NFATc1) or LPS stimulation (NF-kB). NFATc1 and NF-kB were immunostained with Alexa Fluor 594 (red) and nuclei were stained with DAPI (blue). (**d**) NO and TNF-α release in N9 cells pre-treated with 5 µM cyanidin or cyanidin-3-*O*-glucoside for 6 h before LPS stimulation (NF-kB). Representative images are shown, and the values represent the mean ± SEM of at least three biological replicates, ** *p* < 0.01, *** *p* < 0.001.

Cyanidin-hexoside was one of the potential hits selected for anti-inflammatory properties. As a proof of concept, we first evaluated the protective activity of cyanidin-3-*O*-glucoside, the most common cyaniding-hexoside in the *Rubus* species, in the Crz1 activation model. Pelargonidin-3-*O*-glucoside, another common anthocyanin hexoside present in the studied species, was used for comparative purposes. Only cyanidin-3-*O*-glucoside, but not pelargonidin-3-*O*-glucoside, decreased Crz1 activation, validating the prediction from the runs test (Figure 5a). Remarkably, cyanidin aglycone caused similar protection levels to cyanidin-3-*O*-glucoside, whereas pelargonidin aglycone did not, suggesting that the cyanidin moiety is the protective structure.

In a conserved mechanism to Crz1, activated NFAT translocates into the nucleus of immune cells. Therefore, we tested cyanidin and cyanidin-3-*O*-glucoside bioactivity in microglia cells (the immune cells resident in the brain) after stimulation with ATP as a pro-inflammatory insult. The anti-inflammatory activity of both compounds was evaluated in the N9 microglia cell line by following NFATc1 subcellular localization in cells exposed to cyanidin and cyanidin-3-*O*-glucoside before ATP insult. As shown in Figure 5b, NFATc1 was dispersed throughout the cells in control cells, and treatment with ATP led to its nuclear accumulation which is accompanied by cell morphological changes associated with microglia activation. Pre-treatment with both cyanidin-3-*O*-glucoside and cyanidin aglycone prevented NFATc1 accumulation in the nucleus, validating the results obtained using the yeast model of Crz1 activation.

The NF-kB pathway represents a central pathway in inflammatory responses, and the NF-kB regulatory activity is also controlled at the level of subcellular localization. Thus, the anti-inflammatory potential of cyanidin and cyanidin-3-*O*-glucoside towards p65, a subunit of the NF-kB complex, was further investigated in cells pre-treated with both compounds before stimulation with LPS. Similar to NFATc1, both compounds prevented p65 nuclear accumulation (Figure 5c). Notably, only cyanidin aglycone mediated a significant decrease of nitric oxide levels and TNF-α release (Figure 5d,e).

## 4. Discussion

### 4.1. Cell-Based Assays for Discovery of Health-Promoting Attributes in the Rubus Germplasm

Soft fruit from the *Rubus* genus have been described as important sources of phenolic compounds with health-promoting activities. This study describes an integrated approach combining the power of metabolomics with the exploitation of a facile, reliable and cost-effective yeast-based discovery platform. The aim was to mine the diversity of (poly)phenolic compounds in selected berry germplasm of cultivated, wild, and underutilized *Rubus* species for bioactives able to modulate pathological processes associated with major chronic diseases. We have previously used a similar approach to systematically analyze *Rubus idaeus* (var. Prestige) extracts for the identification of bioactive compounds conferring protection against HD. The extract was fractionated, re-tested in the yeast platform; salidroside—a glycosylated phenol—was identified as the bioactive compound [13].

In this study, the untargeted metabolomics analysis identified the 15 most chemically diverse polyphenol-enriched extracts from a *Rubus* germplasm collection composed of 35 species/cultivars. With a minimum number of bioactivity screening efforts in the yeast-based discovery platform, this approach fast-tracked the identification of *R. genevieri* as novel source of potential bioactive compounds for ALS and *R. occidentalis* (var. Huron) conferring potential anti-cancer and anti-inflammatory properties, among other bioactivities indicated in Table 1.

Mechanistic studies were performed and suggest that *R. genevieri* (poly)phenolics led FUS to be trapped into insoluble intracellular structures thereby reducing its pathological activity. This is a well-known route of cellular protection against the toxic effect of several oligomerization-prone proteins described for several diseases [40]. It remains to identify the major molecular players in this process and to evaluate whether this mechanism is conserved in higher eukaryotes.

One-third of human tumors are driven by RAS mutations, especially the KRAS isoform [41], whereas approximately 8% of tumors express an activated form of BRAF [42], accounting together for almost 40% of cancers. Given the impact of deregulated RAS–RAF–MEK–ERK signaling cascade in cancer, the identification of compounds targeting its components has improved clinical outcomes [43]. The model used in the present study relies on the expression of mutant versions of *RAS* and *RAF* genes, encoding hyperactivated proteins of the RAS–RAF–MEK–ERK cell proliferation signaling pathway, in human cells. *Rubus occidentalis* (var. Huron) extracts were identified as a potent inhibitor of RAS/RAF interaction, thus potentially modulating cell proliferation in contexts of dysregulated signaling in cancer. Curiously, protection against pathological cancer processes was concentration dependent and exhibited a hormetic pattern with concentrations higher than 250 µg GAE/mL exerting the opposite effect, i.e., inducing the interaction between HRAS/BRAF above the levels observed for the untreated condition via a mechanism that still remains to be elucidated.

Chronic activation of immune responses is a common link between neurodegeneration and cancer [44]. Therefore, the identification of molecules targeting inflammatory process may impact both pathologies. Modulation of inflammatory processes is driven by persistent activation of key transcription factors, such as NFAT and NF-κB, which in turn upregulate transcription of pro-inflammatory genes creating a positive feedback loop further amplifying initial stimuli. *CRZ1* is the yeast homologue of NFAT, a transcription factor controlling inflammatory responses in humans. Similarly to NFAT, Crz1 regulation is modulated by the calcium (Ca^2+^)-signaling pathway, which culminates in calcineurin (CaN) activation by calmodulin, Crz1 dephosphorylation, and nuclear translocation [29,32]. The same concentration range of *R. occidentalis* (var. Huron) polyphenol-enriched extracts driving potential anti-cancer effects also mediated the anti-inflammatory properties of this extract. A mechanistic insight into the molecular targets of *R. occidentalis* (var. Huron) (poly)phenols was obtained by showing that it inhibits Crz1 nuclear accumulation thereby preventing the expression of the reporter gene *lacZ* as well as its endogenous targets genes.

### 4.2. Exploring Statistical Tools to Expedite Compounds Bioactivity Identification

Discrete differences observed among the metabolite profiles of the extracts analyzed may be responsible for the bioactivity against a range of different disease processes. The literature presented in the last decade clearly indicates that the potential therapeutic effect of some phytochemical components of fruit is determined by qualitative compositional differences and gross measurements, such as total phenol content and total antioxidant potential, do not fully explain the bioactivity of some extracts [45]. While the major phytochemical components present in the extracts are usually well characterized, there is a limited amount of research on the bioactivity associated with the minor phytocomponents. To ensure the optimization of the discovery of novel bioactives, it is of paramount importance that the metabolomics approach used should be untargeted thereby avoiding introducing bias into the analysis.

A traditional approach for the discovery of novel components from bioactive extracts is an iterative process which includes the fractionation of bioactive extracts, re-testing the bioactivity of the various fractions, and subsequent fractionation and testing until a single component is isolated. This is an extremely labor- and time-intensive process with a number of pitfalls often including the lack of available material for performing an extensive number of iterations and the loss of potential synergistic effects. The statistical approach (i.e., the runs test) used in this resulted in the identification of 15 compounds with potential bioactivities for 4 disease models.

A total of three compounds had significance for Huntington’s disease (HD) model including quercetin 3-*O*-glucuronide and a triterpenoid isomer. The supplementation of rodent models with quercetin derivatives attenuated symptoms of HD [46], while recent in vitro and in vivo evidence indicated that quercetin-3-*O*-glucuronide modulated neurogenesis [47]. Additionally, triterpenoids such as celastrol, onjisaponin B, and ginsenosides from various plant sources have provided encouraging evidence of their preclinical efficacy in modulating HD [48].

The runs test for the ALS model resulted in the identification of a unique significant molecular feature tentatively identified as a leucine isomer. Interestingly, ALS has been associated with abnormal glutamate metabolism, and since branched-chain amino acids (BCAAs; e.g., l-leucine, l-isoleucine, and l-valine) can modify glutamate metabolism, these have been evaluated with respect to their therapeutic potential [49]. However, clinical studies have provided conflicting evidence with some studies reporting negative effects or a lack of efficacy [49,50,51], whereas others reported amelioration of ALS symptoms [52,53]. More recently, in vivo preclinical evidence suggests that BCAA, tryptophan, and particularly arginine and proline metabolic pathways are associated with disease progression [54].

The runs test for the RAS/RAF interaction model (cancer model) indicated a total of nine compounds with potential bioactivities that included (−)-epicatechin, various anthocyanins, an unidentified triterpenoid, a hydroxysphingosine isomer, and a benzoic acid di-hexoside. The potential of berry extracts to modulate cancer disease progression have been shown both via in vitro and in vitro models and anthocyanins have been found to be major contributors towards inhibiting cell proliferation and inducing apoptosis [55]. Dietary triterpenoids, which are often found in *Rubus* species, are able to survive in vivo digestion [56] and have shown promising results in pre-clinical trials in colorectal cancer models [57]. Interestingly, (−)-epicatechin has been found to provide synergistic effects in modulating growth and apoptosis in human cancerous cell cultures when combined with other phytochemicals such as curcumin [58] and EGCG [59], whereas epidemiological evidence found association between dietary intake of catechin-derived compounds and reduced colorectal cancer incidence [60,61]. Finally, MS-based fingerprint analyses of biofluids have reported significant lower levels of hydroxysphingosine in the plasma of patients with multiple myeloma, chronic lymphocytic leukemia [62], and urine of patients with prostate cancer [63] in comparison with control healthy individuals.

### 4.3. Identification of a Single Compound—Cyanidin—with Anti-Inflammatory Properties towards NFAT and NF-kB Transcription Factors

The runs test of the Crz1 activation model (inflammation model) indicated two potential bioactive compounds including an anthocyanin hexoside. Anthocyanins have been associated with anti-inflammatory properties in various in vitro and in vivo studies [64], whereas epidemiological evidence has suggested that higher anthocyanin intakes were also associated with increased anti-inflammatory effects [65]. Interestingly, anthocyanin-rich fractions of *Rubus* fruits have also shown anti-inflammatory properties in vitro [66]. In our study, we validated the bioactivity of cyanidin-3-*O*-glucoside for inflammation in yeast and mammalian cells models whereas no activity was found for pelargonidin-3-*O*-glucoside. In addition, our findings unveil the health-promoting attributes of cyanidin-3-*O*-glucoside not only for the target identified in the yeast discovery platform (NFAT), but also for the canonical inflammatory NF-kB pathway as revealed in mammalian in vitro model. This is in agreement with previous studies showing that cyanidin 3-*O*-glucoside exerts inhibitory roles towards the NF-kB proinflammatory pathway in several cell models [67,68,69,70,71]. As for NFAT, little is known, with little evidence indicating that cyanidin 3-*O*-glucoside downregulates NFATc1 thereby inhibiting RANKL-mediated osteoclastogenesis [72]. In contrast, we did not report a significant effect of pelargonidin and pelargonidin 3-*O*-glucoside despite evidence of its role in the inhibition of the NF-kB pathway [73]. The cellular assays extended the predictions made by the runs test and proved that the cyanidin moiety, rather than cyanindin-3-*O*-glucoside, was responsible for the detected bioactivity. This finding illustrates the power of combining metabolomics, in silico analysis, and cellular assays for the identification of single bioactive compounds.

As mentioned before, this study was designed to fast-track potential bioactives to be further explored in more robust pre-clinical models. As such, and also taking into account the limited uptake of certain compounds due to the yeast cell wall, the criteria was using non-toxic concentrations of (poly)phenol-enriched extracts for bioactivity determination. Once a single compound was identified —the cyanidin— and the bioactivity confirmed in yeast, physiologically relevant concentrations (5 µM) [74] were tested in mammalian cell models for study validation in a nutritional point of view.

In brief, our approach revealed potential bioactive compounds for pathological processes associated with redox-related diseases. With regard to cyanidin, the protective effect was validated at levels in the range of described bioavailable concentrations. However, even in the scenario of a compound fails to be active under concentrations in the physiological range, nutraceutical/therapeutical applications could be ensured by the development of formulations for controlled delivery to target tissues

## 5. Conclusions

This study described the use of an integrative approach, combining the power of metabolomics, cellular assays and potent statistical analysis, to identify novel health-promoting attributes in underexplored (poly)phenol sources. The rationale involved the selection of the most chemically diverse samples of an extensive *Rubus* collection followed by the determination of health-promoting activities using a SMART discovery platform.

Overall, the study allowed the identification of (poly)phenol-enriched extracts and single compounds from *Rubus* modulating pathological processes of redox-related diseases responsible for major societal and economic impacts as well as provided some clues regarding the possible molecular mechanisms underlying their protective activity. Our objective was to deliver novel plant (poly)phenolic bioactives with the potential to be exploited either in food engineering and in the pharmaceutical and biotechnological sector as nutraceutical/therapeutic alternatives for redox-related chronic diseases. Of course, in therapeutic applications, development of formulations for controlled delivery to target tissues should further be developed to ensure that physiologically relevant concentrations of bioactive compounds reach their target sites.

Although the number of novel plant (poly)phenolics with potential bioactivity is limited, we tentatively identified several phytochemicals in *Rubus*, such as triterpenoids, benzoyl di-hexoside, hydroxysphingosine, and a leucine isomer, which have not been extensively studied such as *Rubus*-derived bioactive compounds. Interestingly, (−)-epicatechin has previously been described as possessing synergistic effects [58,59], and while our model does not tackle synergistic and antagonistic effects, it is possible that significant results from the runs test could be associated with synergistic effects rather than intrinsically high bioactivity.

While more work is necessary in compound annotation, development of the statistical model in order to cope with synergistic and antagonistic effects and validation of bioactivities in advanced models, this report highlights the feasibility of this strategy for the replication and identification of novel bioactive lead molecules from crude extracts from berry fruits.

## Figures and Tables

**Table 1:** Bioactivity of *Rubus* polyphenol-enriched extracts towards pathological processes of redox-related diseases as determined by the estimation of the protection factor.

*Rubus* Samples	Protective Factor in Each Disease Model					
	αSyn Toxicity ^a^	Aβ42 Toxicity ^a^	HTT Toxicity ^a^	FUS Toxicity ^a^	KRAS/RAF Interaction ^b,c^	Crz1 Activation ^b,d^
*R. vagabundus*	0	0	0	0	0	0
*R. brigantinus*	0	0	10.5 ± 10.1	0	0	35.2 ± 13.1
*R. sampaioanus*	0	0	0	0	0	15.7 ± 9.2
*R. genevieri*	0	0	0	39.0 ± 13.9	0	0
*R. hochstetterorum*	0	0	17.0 ± 10.7	0	0	12.2 ± 8.3
*R. henriquesii*	0	0	16.0 ± 10.1	0	0	10.3 ± 2.9
*R. loganobaccus* var. Tayberry	0	0	0	0	52.5 ± 1.2	0
*R. loganobaccus* var. Sunberry	0	90.3 ± 6.2	87.6 ± 30.7	0	0	6.4 ± 2.6
*R. fruticosus* var. Fantasia	0	0	0	0	0	0
*R. fruticosus* var. Ashton cross	0	0	0	0	0	41.2 ± 5.3
*R. idaeus* var. Prestige	0	0	98.7 ± 28.8	49.4 ± 24.0	15.4 ± 2.0	19.5 ± 0.9
*R. idaeus* var. Octavia	12.3 ± 6.6	0	0	22.4 ± 30.0	0	60.8 ± 8.5
*R. occidentalis* var. Huron	0	0	0	0	57.7 ± 1.2	57 ± 4.9
*R. armeniacus* var. Himalayan giant	0	0	0	0	27.1 ± 6.2	46.4 ± 3.4
*Rubus sp.* B14	0	0	0	0	35.9 ± 6.0	0

a—Protection factor (P%) = 100 × ((AUC_sample_ − AUC_disease_)/(AUC_control_ − AUC_disease_)); b—protection factor (P%) = 100 × (100 − (MU_disease_ − MU_sample_)/(MU_disease_ − MU_control_)); c—KRAS/BRAF interaction; d—Crz1 activation; AUC—area under the curve; MU—Miller units. The darker the green, the greater the bioactivity.

## Supplementary Materials

The following are available online at https://www.mdpi.com/2076-3921/9/9/789/s1. Table S1: *Rubus* species/varieties used in this study, Table S2: Significant compounds from runs test for all disease models tested, Table S3: Yeast strains used in this study, Table S4: Plasmids used in this study, Figure S1: PCA analysis of the entire positive mode dataset, Figure S2: PCA analysis of the entire negative mode dataset, Figure S3: Yeast model of Parkinson’s disease, Figure S4: Yeast model of amyotrophic lateral sclerosis, Figure S5: Yeast model of Huntington’s disease, Figure S6: Yeast model of Alzheimer’s disease, Figure S7: Yeast model of RAS/RAF interaction, Figure S8: Yeast model of Crz1 activation, Figure S9: Supplementary data for the cancer (HRAS/KRAS) model, Figure S10: Supplementary data for the Crz1 activation model, Figure S11: Visual representation of the runs test.
