# Supplementary material for: Bioprospection of Natural Sources of Polyphenols with Therapeutic Potential for Redox-Related Diseases
Source: Antioxidants (Basel). 2020 Aug 26;9(9):789. doi: 10.3390/antiox9090789 (PMC7576474; doi:10.3390/antiox9090789)
Supplement: Supplementary file 1 [file antioxidants-09-00789-s001.pdf]

antioxidants

MDPI

Supplementary Material

Bioprospection of natural sources of polyphenols with therapeutic potential for redox-related diseases

Regina Menezes[1,2,3†], Alexandre Foito[4#], Carolina Jardim[2,3], Inês Costa[2,3], Gonçalo Garcia[2,3], Rita Rosado-Ramos[1,2,3], Sabine Freitag[4], Colin James Alexander[5], Tiago Fleming Outeiro[7,8,9], Derek Stewart[4,6], Claudia Nunes Santos[1,2,3*]

Regina Menezes [1,2,3,†], Alexandre Foito [4,†], Carolina Jardim [2,3], Inês Costa [2,3], Gonçalo Garcia [2,3], Rita Rosado-Ramos [1,2,3], Sabine Freitag [4], Colin James Alexander [5], Tiago Fleming Outeiro [6,7,8], Derek Stewart [4,9] and Cláudia N. Santos [1,2,3,*]

1 CEDOC, Chronic Diseases Research Centre, NOVA Medical School / Faculdade de Ciências Médicas, Universidade NOVA de Lisboa, Campo dos Mártires da Pátria, 130, 1169-056 Lisboa, Portugal; regina.menezes@nms.unl.pt (R.M.); rita.ramos@nms.unl.pt (R.R.-R.); (C.N.S.)
2 iBET, Instituto de Biologia Experimental e Tecnológica, Apartado 12, 2781-901 Oeiras, Portugal; cjardim@medicina.ulisboa.pt (C.J.); inescosta.bms@gmail.com (I.C.); ggarcia@campus.ul.pt (G.G.)
3 Instituto de Tecnologia Química e Biológica, Universidade Nova de Lisboa, Av. da República, 2780-157 Oeiras, Portugal
4 Environmental and Biochemical Science Group, The James Hutton Institute, DD2 5DA Dundee, UK; alex.foito@hutton.ac.uk (A.F.); Sabine.Freitag@hutton.ac.uk (S.F.); Derek.Stewart@hutton.ac.uk (D.S.)
5 Biomathematics and Statistics Scotland, Invergowrie, DD2 5DA Dundee, UK; Colin.Alexander@hutton.ac.uk
6 Department of Experimental Neurodegeneration, Center for Nanoscale Microscopy and Molecular Physiology of the Brain, Center for Biostructural Imaging of Neurodegeneration, University Medical Center Göttingen, 37073 Göttingen, Germany; touteir@gwdg.de
7 Max Planck Institute for Experimental Medicine, 37075 Göttingen, Germany
8 Translational and Clinical Research Institute, Faculty of Medical Sciences, Newcastle University, Framlington Place, Newcastle Upon Tyne, NE2 4HH, UK
9 School of Engineering and Physical Sciences, Institute of Mechanical, Process and Energy Engineering, Heriot-Watt University, EH14 4AS Edinburgh, UK; D.Stewart@hw.ac.uk
† These authors contributed equally to this work.
* Correspondence: claudia.nunes.santos@nms.unl.pt

Received: 22 July 2020; Accepted: 18 August 2020; Published: date

INDEX

1. ***Supplementary tables*****.....3**

Table S1. *Rubus* species/varieties used in this study.....3

Table S2. Significant compounds from Runs test for all disease models tested....5

Table S3. Yeast strains used in this study.....7

Table S4. Plasmids used in this study.....8

2. ***Supplementary methods*** **.....9**

2.1 Yeast plasmids and strains.....9

2.2 Yeast growth conditions.....9

2.3 Growth assays.....9

2.4 Growth curve data analysis methods.....9

2.5 Flow cytometry.....9

2.6 Fluorescence microscopy.....10

2.7 Protein extraction and immunoblotting.....10

2.8 β–Galactosidase assays.....10

2.9 Statistical analysis.....10

3. ***Supplementary figures – Part I*** **.....11**

Figure S1. PCA analysis of entire positive mode dataset.....11

Figure S2. PCA analysis of entire negative mode dataset.....12

4. ***Supplementary information*****:** ***SMART discovery platform*****.....13**

Figure S3. Yeast model of Parkinson's disease.....14

Figure S4. Yeast model of Amyotrophic Lateral Sclerosis.....15

Figure S5. Yeast model of Huntington's disease. 16

Figure S6. Yeast model of Alzheimer's disease.....17

Figure S7. Yeast model of RAS/RAF interaction.....18

Figure S8. Yeast model of Crz1 activation.....19

Figure S9. Supplementary data for the cancer (HRAS/KRAS) model.....20

Figure S10. Supplementary data for the Crz1-activation model.....21

5. ***Supplementary figures – Part II*** **.....22**

Figure S11. Visual representation of the Runs test.....22

6. ***References*** **.....23**

1. Supplementary tables

Table S1 I *Rubus* species/varieties used in this study.

Species	Variety	Source Location
Rubus armeniacus Focke [= *R. procerus* auct.])	Himalayan Giant	UK
Rubus bartonii Newton syn. 'Ashton Cross'	Ashton Cross	UK
Rubus fruticosus L. agg.	B13*	UK
Rubus fruticosus L. agg.	B14*	UK
Rubus ursinus x R. armeniacus x R. idaeus	Black Butte	UK
Rubus fruticosus L. *agg.*	Fantasia	UK
Rubus fruticosus L. agg.	Karaka Black	UK
R. ursinus x *R.ursinus* x *R.idaeus* x *R. ursinus* x *R. allegheniensis* x *R. argutus*	Kotata	UK
Rubus ursinus x *R.ursinus* x *R.idaeus*	Ollalie	UK
Rubus idaeus L.	Glen Ample	UK
Rubus idaeus L.	Glen Fyne	UK
Rubus idaeus L.	Malling Admiral	UK
Rubus idaeus L.	Malmer Szedler	UK
Rubus idaeus L.	Octavia	UK
Rubus idaeus L.	Polana	UK
Rubus idaeus L.	Prestige	UK
Rubus idaeus L.	Tulameen	UK
Rubus loganobaccus L.H.Bailey	Nectarberry	UK
Rubus loganobaccus L.H.Bailey	Boysenberry	UK
Rubus loganobaccus L.H.Bailey	Loganberry	UK
Rubus loganobaccus L.H.Bailey	LY59-10*	UK
Rubus loganobaccus L.H.Bailey	Riwaka Choice	UK
Rubus loganobaccus L.H.Bailey	Sunberry	UK
Rubus loganobaccus L.H.Bailey	Tayberry	UK

Rubus occidentalis L.	Black Hawk	UK
Rubus occidentalis L.	Huron	UK
Rubus occidentalis L.	Plum Farmer	UK
Rubus phoenicolasius Maxim.	Wineberry	UK
Rubus brigantinus Samp.	-	PT
Rubus genevieri Boreau.	-	PT
Rubus henriquesii Samp.	-	PT
Rubus hochstetterorum Seub.	-	PT
Rubus sampaioanus Sudre ex Samp.	-	PT
Rubus vagabundus Samp.	-	PT

Table S2 I Significant compounds from Runs test for all disease models tested.

Retention time (min)	Mode	m/z	Adduct/ fragment	Molecular Formula (<5ppm)	Putative ID	ID confidence level[1]	Runs test significance level[2]	Disease model
19.93	Positive	479.0822	$[M+H]^+$	$C_{21}H_{18}O_{13}$	Quercetin 3-O-glucuronide	1	**	HTT toxicity
23.58	Negative	625.0597	-	N/A	Unknown	4	*	
24.50	Positive	503.3365	$[M+H]^+$	$C_{30}H_{46}O_6$	Hydroxy-oxopomolic acid isomer	3	**	
2.48	Positive	132.1020	$[M+H]^+$	$C_6H_{13}NO_2$	leucine or leucine-isomer	2	*	FUS toxicity
13.38	Negative	323.0977	$[M-Benzoic acid-H]^-$ in-source fragment	$C_{19}H_{26}O_{12}$	Benzoyl-dihexoside	3	*	KRAS/BRAF interaction
13.40	Positive	464.1815	$[M+NH4]^+$			3	*	
13.40	Positive	267.0897	$[M-gluc+H]^+$ in-source fragment			3	*	
13.41	Positive	105.0378	$[M-2glucose+H]^+$ in-source fragment			3	*	
14.55	Positive	291.0864	$[M+H]^+$	$C_{15}H_{14}O_6$	(-)-Epicatechin	1	*	
14.55	Negative	289..0718	$[M-H]^-$			1	*	
14.63	Positive	381.1148	-	N/A	Unknown	4	*	
15.26	Positive	613.1682	C_{13} Isomer of 611.1632	$C_{27}H_{31}O_{16}^+$	Cyanidin 3-O-Sophoroside	1	**	
15.33	Negative	743.2037	$[M-H]^-$	$C_{32}H_{40}O_{20}$	Unknown	4	*	
17.04	Positive	579.1709	M^+	$C_{27}H_{31}O_{14}^+$	Pelargonidin 3-O-rutinoside	1	*	

17.26	Positive	609.1812	M^+	$C_{28}H_{33}O_{15}^+$	Peonidin 3-O-rutinoside	1	*	
23.52	Positive	471.3476	$[M+H]^+$	$C_{30}H_{46}O_4$	Unidentified Triterpenoid	3	*	
36.35	Positive	316.2849	$[M+H]^+$	$C_{18}H_{37}NO_3$	Hydroxysphingosine	2	*	
14.65	Positive	897.2101	$[2M^+-H]^+$	$C_{21}H_{21}O_{11}^+$	Cyanidin-hexoside	3	*	Crz1 activation
20.56	Positive	667.2984	$[M+H]^+$	$C_{33}H_{46}O_{14}$	Unknown	4	**	

[1] The Metabolomics Standards Initiative [1] defined four levels of metabolite identification confidence to which the manuscript has adhered to. Confidently identified compounds are classified as a level 1 and require evidence based on two or more orthogonal properties with an authentic chemical standard analyzed under identical analytical conditions. Putatively annotated compounds are classified as level 2 and are based upon physicochemical properties and/or spectral similarity with public commercial spectral libraries, without reference to authentic chemical standards. Putatively annotated compound classes are categorized as level 3, and are based upon characteristic physicochemical properties of a chemical class of compounds, or by spectral similarity to know compounds of a chemical class. Unknown compounds are classified as level 4 and although they remain unidentified and unclassified, these metabolites can still be differentiated and quantified based upon spectral data.;
[2]Statistical significance level **p-value* <0.05, ***p-value*<0.01

Table S3 I Yeast strains used in this study.

Strain	Genotype	Source or Reference
W303-1A_Syn	*MAT*a *can1-100 his3-11,15 leu2-3,112 ade2-1 GAL1pr-syn WT::TRP1 GAL1pr-syn WT::URA3*	[2]
W303-1A_TU*	*MAT*a *can1-100 his3-11,15 leu2-3,112 ade2-1 TRP1 URA3*	[2]
W303-1A_FUS	*MAT*a *can1-100 ura3-1 his3-11,15 leu2-3,112 ade2-1 GAL1pr-FUS::TRP1*	This study
W303-1A_T*	*MAT*a *can1-100 ura3-1 his3-11,15 leu2-3,112 ade2-1 TRP1*	This study
BY4741_erg6	*MATa his3Δ1 leu2Δ0 lys2Δ0 ura3Δ0 YBR082c::kanMX4*	EUROSCARF **
SKY197	*MATα ura3 his3 trp1 1LexAop-LEU2 λcIop-LYS2 pdr1::GAL1pr-HXT9 pdr3::GAL1pr-HXT11*	[3]
YAA5	*MATα his3 leu2 lys2 ura3 aur1::AUR1-C-4xCDRE-lacZ*	[4]
YAA6_crz1*	*MATα his3 leu2 lys2 ura3 YNL027W::HIS3MX4 aur1::AUR1-C-4xCDRE-lacZ*	[4]
YAA7_cnb1*	*MATα his3 leu2 lys2 ura3 YKL190W:: kanMX4 aur1::AUR1-C-4xCDRE-lacZ*	[4]
YAA3	*MATα his3::CRZ1-GFP-HIS3 leu2 ura3 met15*	[4]

*****Control strains, ****EURO**PEAN **S**ACCHAROMYCES **C**EREVISIAE **AR**CHIVE FOR **F**UNCTIONAL ANALISYS (http://web.uni-frankfurt.de/fb15/mikro/euroscarf/)

Table S4 I Plasmids used in this study.

Plasmid	Plasmid features	Source or Reference
pAG304_GAL1pr-FUS	Integrative	[5]
pAG304_ GAL1pr-ccdB*	Integrative	[5]
pYES_GAL1pr-FUS-GFP	2μ	[5]
pYES_CT*	2μ	[5]
p426_GAL1pr-FLAG-HTTp103-GFP	2μ	This study
p426_GAL1pr-GFP-AB42	2μ	This study
p426*	2μ	ATCC® 87341™ **
p425_GAL1pr-GFP-AB42	2μ	This study
p425*	2μ	ATCC® 87339™ **
pGKS5_ADH1pr-acKRAS V12	2μ	[3]
pGKS5_ADH1pr-HRAS V12	2μ	[3]
pGKS5*	2μ	[3]
pJG4-5_ GAL1pr-BRAF	2μ	[3]
pJG4-5*	2μ	[3]

* Empty plasmids used as controls, ** **A**merican **T**ype **C**ulture **C**ollection

2. *Supplementary methods*

2.1 Yeast plasmids and strains

All plasmids and strains used in this study are listed in **Table S1 and S2**. To construct p426_GAL1pr-FLAG-HTT103Q-GFP, the sequence *GAL1pr-FLAG-HTTp103-GFP* from p425GAL1_HTT103Q was amplified by PCR and cloned into the p426 vector using the In-Fusion Cloning kit (TAKARA Clontech). p426_GAL1pr-GFP-Aβ42 was generated by inserting the sequence GFP-Aβ42, obtained by the double digestion of p416_GPDpr-GFP-Aβ42 with *Bam*HI/*Sma*I, into the p426 vector. p425_GAL1pr-GFP-Aβ42 was generated by subcloning the sequence GAL1pr-GFP-Aβ42 into the *Sac*I/*Hin*dIII restriction sites of p425 vector.

2.2 Yeast growth conditions

SC medium containing 1% raffinose was used for growth of PD and ALS integrative yeast models. Synthetic dropout $CSM_{\text{-URA}}$ medium containing 1% raffinose was used for growth of ALS episomal yeast model. Synthetic dropout $CSM_{\text{-LEU,}}$ $CSM_{\text{-LEU-URA}}$ and $CSM_{\text{-HIS-URA-TRP}}$ media supplemented with standard concentrations of the required amino acids and containing 1% raffinose, were used for growth of HD, AD and RAS-RAF-interaction yeast models, respectively. Growth of Crz1-activation yeast model was performed in SC medium containing 2% glucose and Crz1 activation was induced with 1.8 mM $MnCl_2$. Cells cultures were prepared as described in **Materials and Methods**.

2.3 Growth assays.

Growth assays were carried out as described in **Materials and Methods** section.

2.4 Growth curve data analysis methods

Raw data were exported from Excel and read into R software for plots construction, calculation of growth parameters and performance of statistical analysis to compare curves (our unpublished data). Briefly, values of optical densities at 600 nm (raw OD) were read for 9 replicates, and the corresponding blank values. The procedure of Toussaint and Conconi [6] was then implemented: raw ODs were subtracted by the correspondent blank value to give the corrected OD values; whenever corrected OD values were negative (which implies that the OD was smaller or equal to the correspondent blank value), a corrected OD of 0.001 was used; corrected ODs were divided by the minimum OD and then transformed applying the natural logarithm (giving lnODs). Corrected OD values divided by the minimum OD were represented graphically. To calculate the growth parameters we used Adjustment of a model-free spline (nonparametric) and Model fitting (parametric) approaches.

2.5 Flow cytometry

Growth assays were carried out as described in **Materials and Methods**. To analyze cell viability with PI, cells were incubated with 20 μg/mL of PI for 30 min at 30ºC protected from light. FCM was performed in a FACS BD Calibur, equipped with a blue solid state laser (488 nm), green fluorescence channel 530/30 nm, and orange red fluorescence channel 610/20 nm.

2.6 Fluorescence microscopy

Fluorescence microscopy was carried out as described in **Materials and Methods**.

2.7 Protein extraction and immunoblotting

Protein extraction and immunoblotting were carried out as described in **Materials and Methods**, using the TCA protein extraction protocol.

2.8 β–Galactosidase assays

β–Galactosidase assays were carried out as described in **Materials and Methods.** Monitoring of Crz1 activation in solid medium was performed similarly with the exception that cells were patched onto solid glucose medium supplemented or not with $MnCl_2$ for 90 min before the overlay procedure.

2.9 Statistical analysis

Statistical analyses were carried out as described in **Materials and Methods.**

3. Supplementary figures – Part I

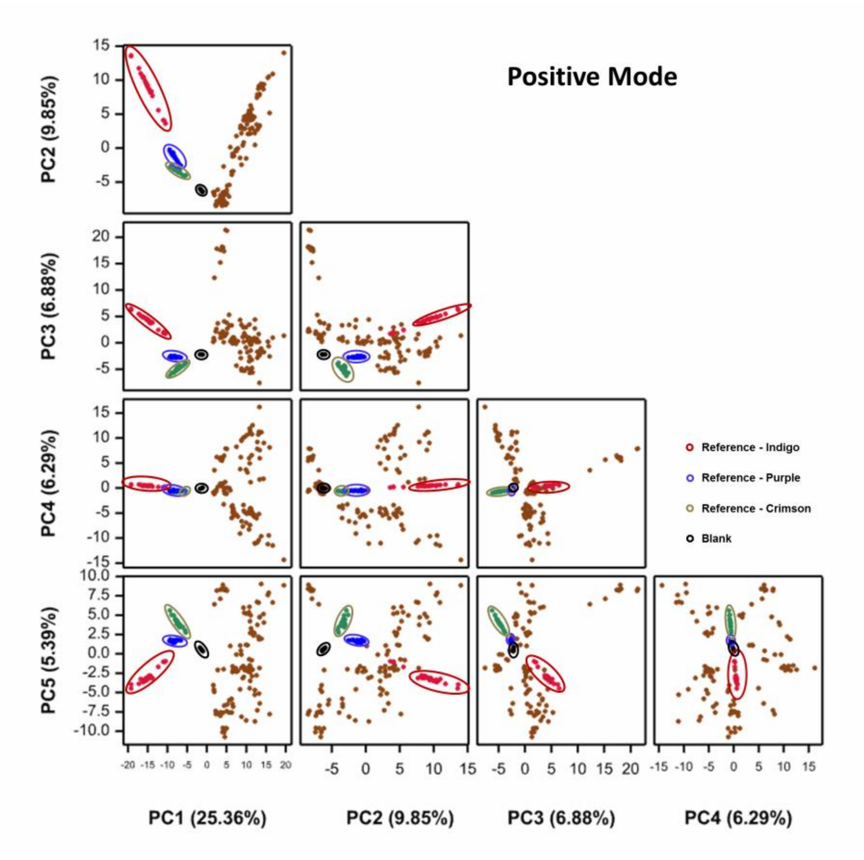

Figure S1. PCA of the correlation matrix of the entire positive mode dataset, including blanks, QC samples (*S. lycopersicum* Crimson, Indigo [7] and Purple [8]) and *Rubus* samples. Principal components 1, 2, 3, 4 and 5 explain 25.36, 9.85, 6.88, 6.29 and 5.39% of the variation, respectively. The PCA plots indicate a clear distinction between the samples and QCs and blanks. The QCs consist of tomato juice from tomatoes expressing different classes of (poly)phenols thereby confirming that the analytical method utilized in this study successfully distinguishes *Rubus* (green) from non-*Rubus* material and different tomato juices based on their (poly)phenolic profiles.

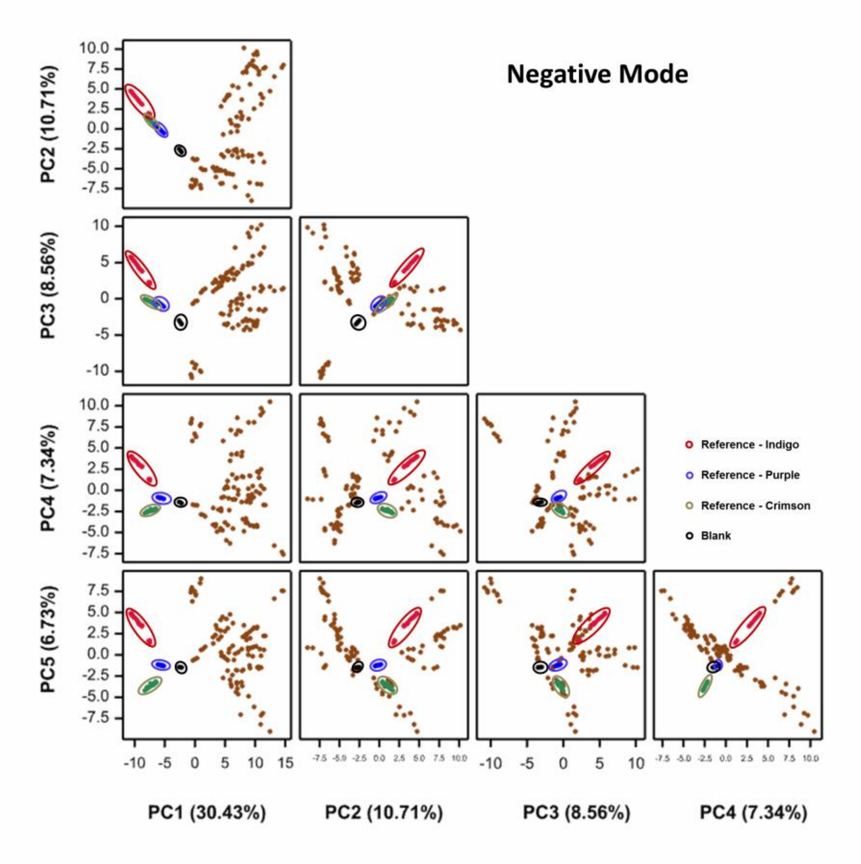

Figure S2. PCA analysis of entire negative mode dataset, including blanks, QC samples (*S. lycopersicum* Crimson, Indigo [7] and Purple [8]) and *Rubus* samples. Principal components 1, 2, 3, 4 and 5 explain 30.43, 10.71, 8.56, 7.34 and 6.73% of the variation, respectively. The PCA plots indicate a clear distinction between the samples and QCs and blanks. The QCs consist of tomato juice from tomatoes expressing different classes of (poly)phenols thereby confirming that the analytical method utilized in this study successfully distinguishes *Rubus* (green) from non-*Rubus* material and different tomato juices based on their (poly)phenolic profiles.

3. Supplementary information: SMART discovery platform

A yeast-based screening platform was used for the identification of *Rubus* bioactivities. We used various yeast models of redox-related neurodegenerative diseases (NDs) based on the expression of human genes associated with different NDs, (*SNCA* [2], *FUS*/*TLS* [5], *HTTpQ103* [9] or *Aβ42* [10]) fused to GFP (**Figures S3a-S6a**). Upon induction of expression of each protein with galactose, the growth of yeast cells was impaired (**Figures S3b-S6b, S3c-S6c**). Growth data was modeled using nonlinear parametric regression to estimate the growth parameters (final biomass, maximum growth rate, lag time, doubling time and area under curve – AUC), as well as the percentage of protection (with 95% confidence intervals) (**Figures S3c-S6c**). The AUC parameter was used to calculate the protection factor of *Rubus* extracts towards each disease pathological process.

Protein expression, inferred by the increase in GFP fluorescence signal (**Figures S3d-S6d**) was accompanied by an increase of propidium iodide-permeable cells in the PD model, indicating also cytotoxicity that was associated with the accumulation of protein inclusions (**Figures S3e-S6e**). We used the various yeast models for the identification of bioactive extracts/compounds that interfered with specific cellular pathologies associated with NDs.

In addition, we also included models of cancer-associated cellular pathologies in the SMART discovery platform. These consisted in the expression of mutant versions of *RAS* and *RAF* genes, encoding hyperactivated proteins of the RAS–RAF–MEK–ERK cell proliferation signaling pathway, in human cells. The yeast two-hybrid approach was previously shown to be an effective tool to address the pathological interaction between RAS and RAF, upstream of the MEK–ERK signaling pathway. In the RAS/RAF yeast model, KRAS and HRAS isoforms were fused to λCI DNA binding domain and its expression controlled by the constitutive *ADH1* promoter whereas BRAF was expressed as fusion with the bacterial B42 activation domain under the control of the galactose-inducible *GAL1* promoter [3]. Protein interaction was assessed by the activation of *lacZ* and *LYS2* genes (**Figure S7a**), through the measurement of *β*-galactosidade activity (**Figures S7b,c**) and cellular growth in media devoid of lysine (**Figures S7d,e**). This simple system greatly facilitates the identification of bioactive molecules potentially inhibiting RAS/RAF pathological interactions in large collections.

As a common link between neurodegeneration and cancer, inflammation models were also included in the discovery platform. Crz1 is the yeast homologue of NFAT, a transcription factor controlling inflammatory responses in humans. Similarly to NFAT, Crz1 regulation is modulated by the calcium (Ca^{2+})-signaling pathway, which culminates in calcineurin (CaN) activation by calmodulin, Crz1 dephosphorylation and nuclear translocation [11,12]. The yeast Ca^{2+}/CaN/Crz1 reporter strain encodes *lacZ* under the control of a promoter bearing Crz1 binding sites (Crz1-*lacZ* model) [4] (**Figure S8a**), representing an easy tool to assess Crz1 activation through the measurement of *β*-galactosidade activity [12] (**Figure S8c**). An additional strain, encoding the fusion *CRZ1*-GFP driven by the native *CRZ1* promoter (**Figure S8b**) allowed assessment of Crz1 nuclear accumulation in cells with disturbed Ca^{2+} cytosolic levels [4] (**Figure S8d**). Given the evolutionary conservation of NFAT and Crz1 activation mechanisms, reinforced by the conserved activity of FK506 and cyclosporin immunosuppressants in both yeast and humans [13], the yeast Ca^{2+}/CaN/Crz1 reporter assay represents an easy and reliable tool to identify small molecules with potential to attenuate NFAT-mediated inflammatory responses.

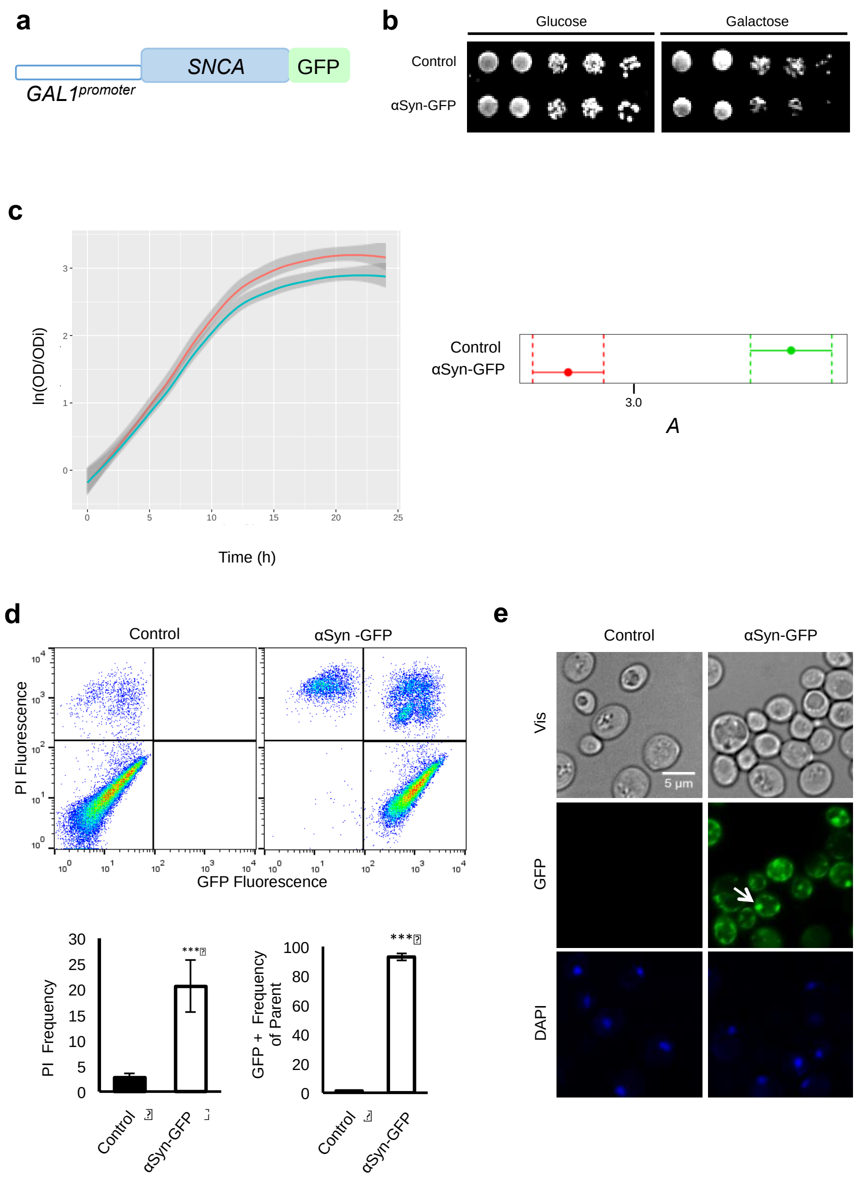

Figure S3. Yeast model of Parkinson's disease. (**a**) Schematics of *SNCA* construct indicating the yeast *GAL1* promoter and the chimeric fusion *SNCA*-GFP. W303-1A recombinant cells expressing two copies of *SNCA*-GFP were pre-grown in SC raffinose medium and cells containing the empty vectors were used as control. (**b**) Cell viability assessed by phenotypic growth assays on SC glucose and SC galactose media. (**c**) Growth curves of cells diluted in SC galactose medium and incubated for 24 h. Growth parameters were estimated as described in 2.4. 95% confidence intervals for the final biomass (*A)* are shown as the growth parameter most affected in this disease model (right panel). (**d**) Cell viability and αSyn expression evaluated by PI vs αSyn-GFP fluorescence, respectively, assessed by FCM (upper panel). The percentage of αSyn-GFP-positive and PI-positive cells is shown (lower panel). (**e**) Fluorescence microscopy images of cells induced with galactose, indicating αSyn intracellular inclusions. Representative images are shown and the values represent the mean ± SEM of at least three biological replicates, ***$p < 0.001$.

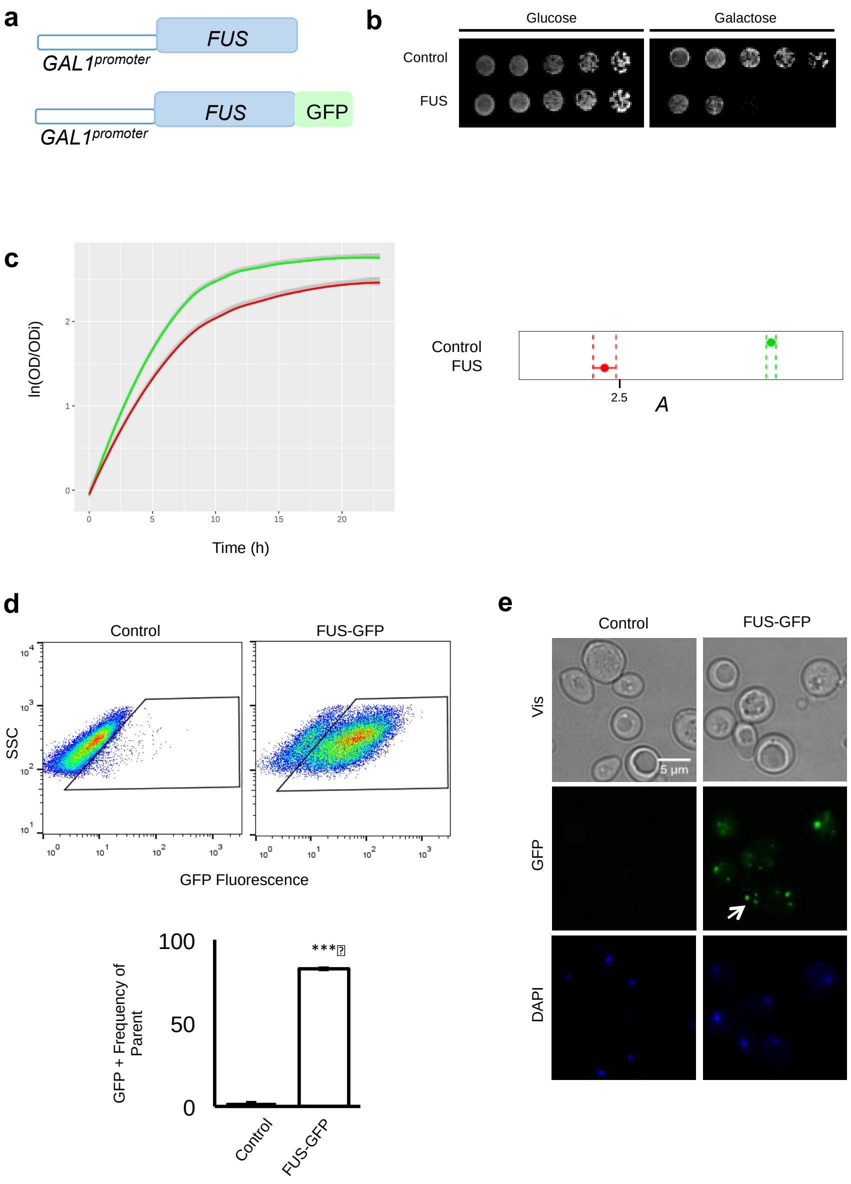

Figure S4. Yeast model of Amyotrophic Lateral Sclerosis. (**a**) Schematics of *FUS* constructs indicating the yeast *GAL1* promoter and the constructs, fused or not with GFP. W303-1A recombinant cells expressing FUS were pre-grown in SC raffinose medium and cells containing the empty vector were used as control. (**b**) Cell viability assessed by phenotypic growth assays on SC glucose and SC galactose media. Representative experiments are shown. (**c**) Growth curves of cells diluted in SC galactose medium and incubated for 24 h. Growth parameters were estimated as described in 2.4. 95% confidence intervals for the final biomass (*A)* are shown as the growth parameter most affected in this disease model (right panel). (**d**) FUS expression (SSC vs FUS-GFP fluorescence) assessed by FCM (upper panel). The percentage of FUS-GFP-positive cells is shown (lower panel). (**e**) Fluorescence microscopy images of cells induced with galactose, indicating FUS intracellular inclusions. Representative images are shown and values represent the mean ± SEM of at least three biological replicates, ***$p < 0.001$.

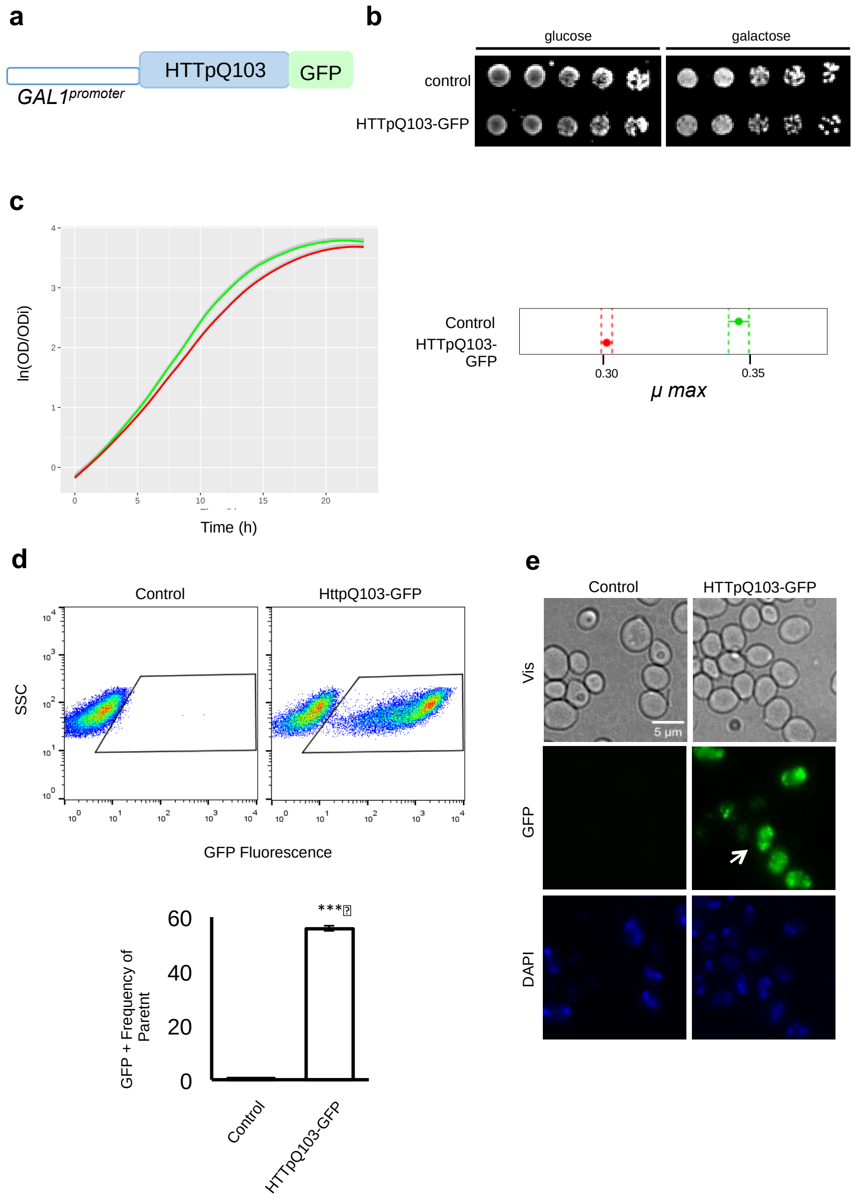

Figure S5. Yeast model of Huntigton's disease. (**a**) Schematics of *HTTpQ103* construct indicating the yeast *GAL1* promoter and the chimeric fusion *HTTpQ103-GFP*. BY4741_*erg6* recombinant cells expressing HttpQ103-GFP from a 2μ vector were pre-grown in SD raffinose medium and cells containing the empty vector were used as control. (**b**) Cell viability assessed by phenotypic growth assays on SD glucose and SD galactose media. (**c**) Growth parameters were estimated as described in 2.4. 95% confidence intervals for the maximum growth rate (μ max) are shown as the growth parameter most affected in this disease model (right panel). (**d**) HttpQ103 expression (SSC vs HttpQ103-GFP fluorescence) assessed by FCM (upper panel). The percentage of HttpQ103-GFP-positive cells is shown (lower panel). (**e**) Fluorescence microscopy images of cells induced with galactose, indicating HttpQ103 intracellular inclusions. Representative images are shown and values represent the mean ± SEM of at least three biological replicates, ***$p < 0.001$.

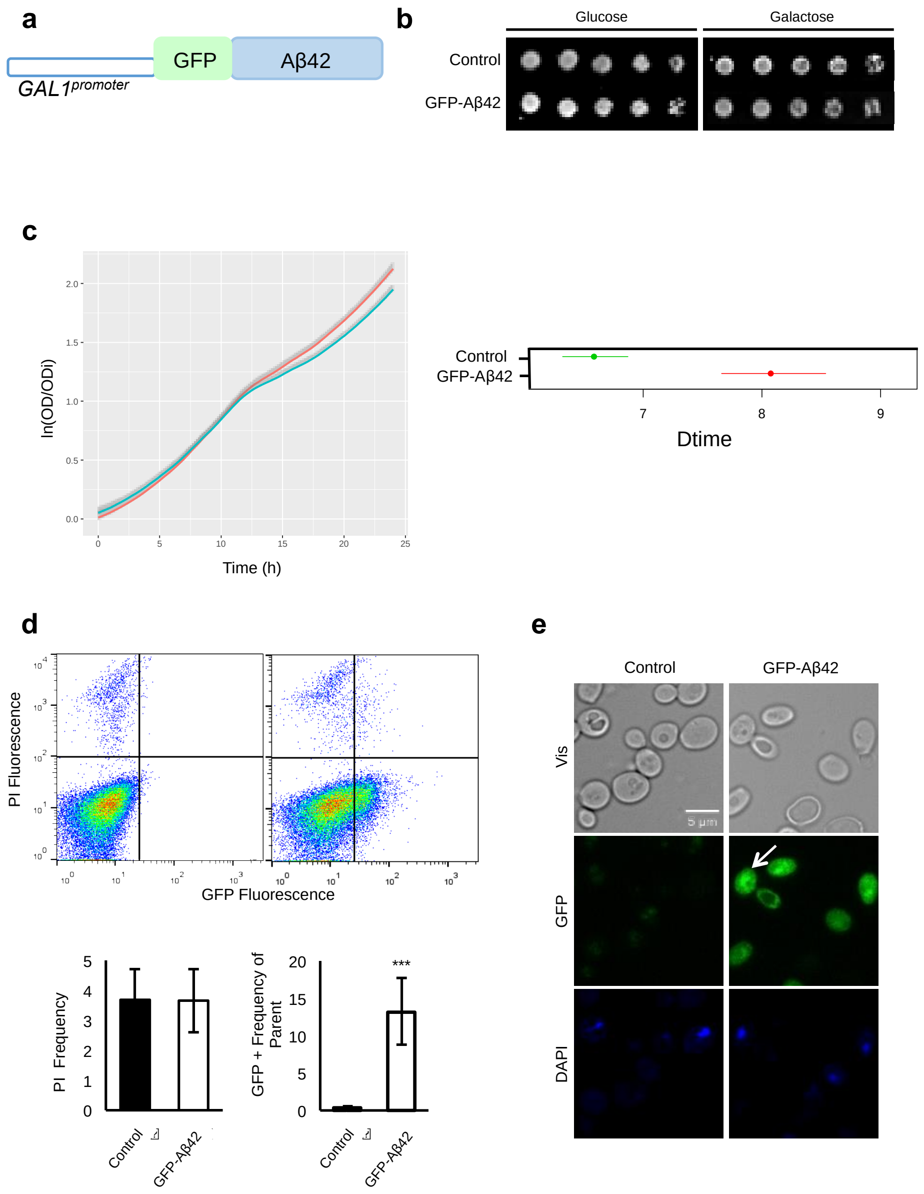

Figure S6. Yeast model of Alzheimer's disease. (**a**) Schematics of *Aβ42* construct indicating the yeast *GAL1* promoter and the chimeric fusion *GFP*-Aβ42. BY4741_*erg6* recombinant cells expressing GFP-Aβ42 from 2μ vectors were pre-grown in SD raffinose medium and cells containing the empty vectors were used as control. (**b**) Cell viability assessed by phenotypic growth assays on SD glucose and SD galactose media. (**c**) Growth curves of cells diluted in SC galactose medium and incubated for 24 h. Growth parameters were estimated as described in 2.4. 95% confidence intervals for the doubling time (Dtime) as the growth parameter most affected in this disease model (right panel). (**d**) Cell viability and Aβ42 expression evaluated by PI vs GFPAβ42 fluorescence, respectively, assessed by FCM (upper panel). The percentage of GFP-Aβ42-positive and PI-positive cells is shown (lower panel). (**e**) Fluorescence microscopy images of cells induced with galactose, indicating Aβ42 intracellular inclusions. Representative images are shown and values represent the mean ± SEM of at least three biological replicates, ***$p < 0.001$.

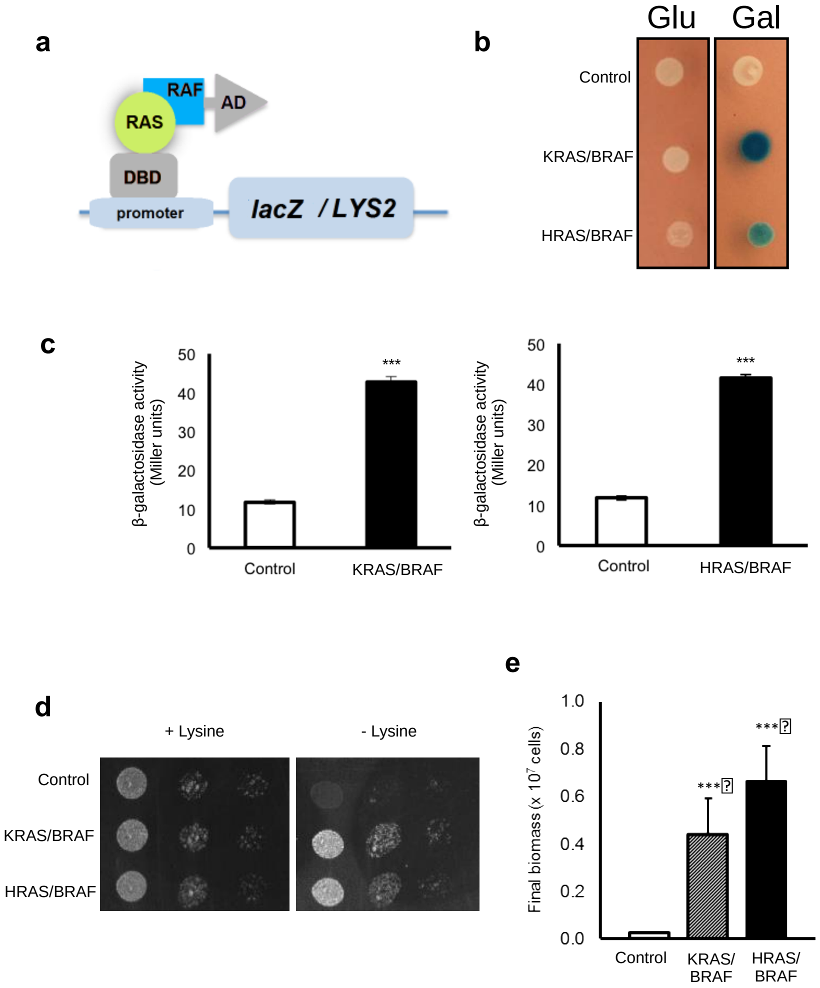

Figure S7. Yeast model of RAS/RAF interaction. (**a**) Schematics of the yeast two-hybrid system used to address RAS/RAF interaction. Constitutively activated versions of KRAS and HRAS human isoforms fused to λCI DNA binding domain (DBD) were co-expressed with human BRAF fused to B42 activation domain (AD) and protein interaction was inferred through the activation of *lacZ* or *LYS2* genes. SKY197 recombinant cells expressing *ADH1pr-λ*CI-RAS and *GAL1pr-B42-BRAF* from 2μ vectors were pre-grown in SD raffinose medium and cells containing the empty vectors were used as control. (**b**) RAS/RAF interaction assessed by monitoring of β-galactosidase activity in SD glucose and SD galactose media using X-Gal. (**c**) RAS/RAF interaction assessed by monitoring of β-galactosidase activity in SD galactose media using ONPG. (**d**) RAS/RAF interaction assessed by phenotypic growth assays on SD galactose medium supplemented or not with lysine. (**e**) RAS/RAF interaction assessed by monitoring the final biomass of cell cultures grown in SD galactose medium without lysine. Representative images are shown and values represent the mean ± SEM of at least three biological replicates, ***$p < 0.001$.

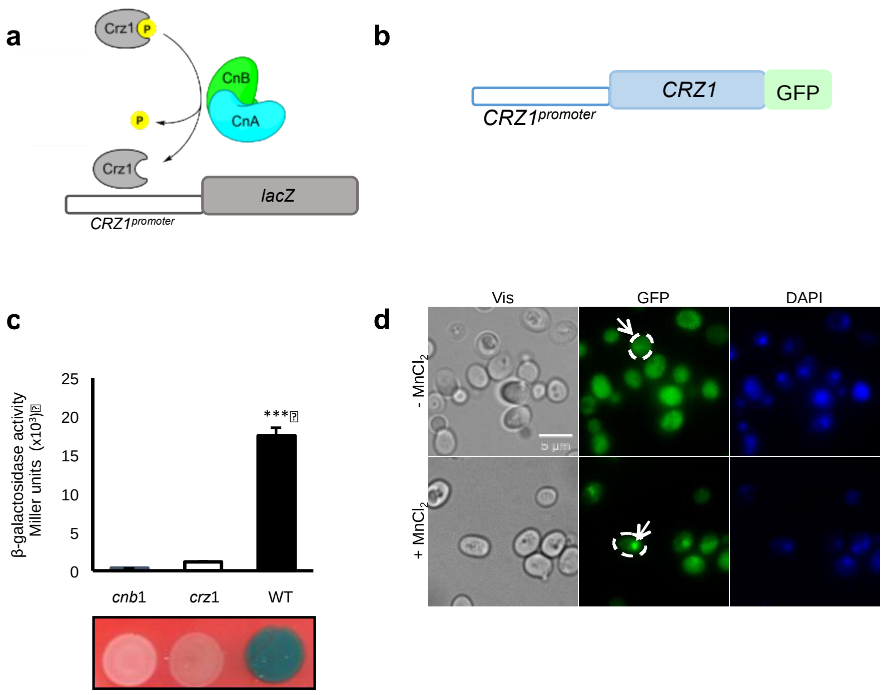

Figure S8. Yeast model of Crz1 activation. (**a**) Schematics of *CRZ1*^{promoter}*-lacZ* reporter system. (**b**) Schematics of *CRZ1* construct indicating the *CRZ1* promoter and the chimeric fusion *CRZ1-GFP*. (**c**) YAA5, YAA7_*cnb1*, and YAA6_*crz1* cells encoding *CRZ1*^{promoter}*-lacZ* were grown in SC glucose medium and Crz1 activation was assessed by monitoring β-galactosidase activity in medium supplemented with 1.8 mM $MnCl_2$, using ONPG (upper panel). X-Gal was used to assess β-galactosidase activity in solid medium (lower panel). (**d**) Crz1 subcellular dynamics evaluated by fluorescence microscopy images in YAA3 Crz1-GFP-expressing cells grown in SC glucose medium supplemented or not with $MnCl_2$. Representative images are shown and the values represent the mean ± SEM of at least three biological replicates, ***$p < 0.001$.

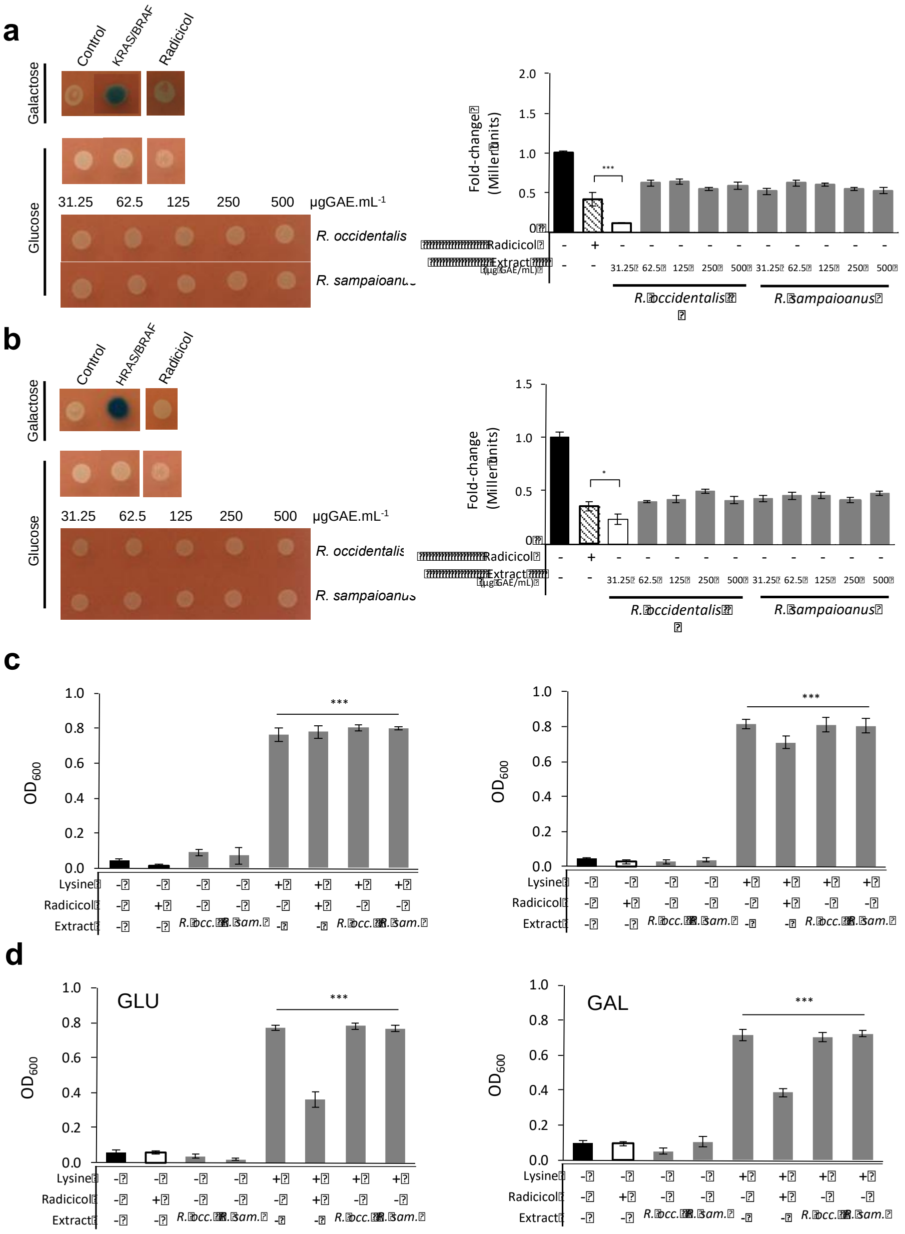

Figure S9. Controls assays of RAS/RAF interaction. Constitutively activated versions of KRAS and HRAS human isoforms fused to λCI DNA binding domain (DBD) were co-expressed with human BRAF fused to B42 activation domain (AD) and protein interaction was inferred through the activation of *lacZ* or *LYS2* genes. Cells were grown in SD raffinose medium prior to experiments and cells containing the empty vectors were use as control. (**a**) KRAS/BRAF and (**b**) HRAS/BRAF interaction assessed by monitoring of β-galactosidase activity in SD glucose media using X-Gal (left panel) and ONPG (right panel). (**c**) KRAS/BRAF (left panel) and HRAS/BRAF (right panel) interaction assessed by monitoring the final biomass of cell cultures grown in SD glucose medium supplemented or not with lysine and 125 μgGAE.mL^{-1} of extracts (**d**) Assessment of the final biomass of cell cultures expressing the control plasmids grown in SD glucose (left panel) or SD galactose (right panel) medium supplemented or not with lysine and 125 μgGAE.mL^{-1} of extracts. Representative images are shown and the values represent the mean ± SEM of at least three biological replicates, *p < 0.05, ***p < 0.001. *R. occ.* – *R. occidentalis; R. sam.* – *R. sampaioanus*

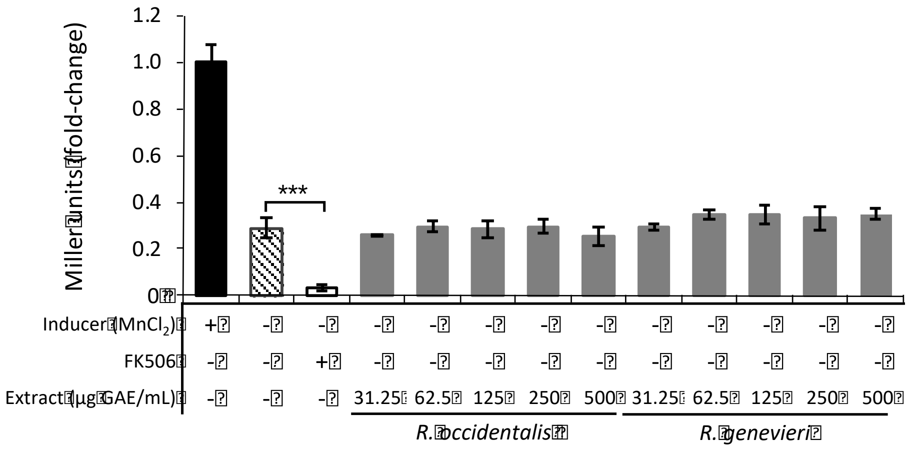

Figure S10. Controls assays of Crz1 activation. YAA5 cells encoding *CRZ1[promoter]-lacZ* were grown in SC glucose medium and Crz1 activation was assessed by monitoring β-galactosidase activity in medium without $MnCl_2$ using ONPG (upper panel). X-Gal was used to assess β-galactosidase activity in solid medium (lower panel). Representative images are shown and the values represent the mean ± SEM of at least three biological replicates, ***$p < 0.001$.

5. *Supplementary figures – Part II*

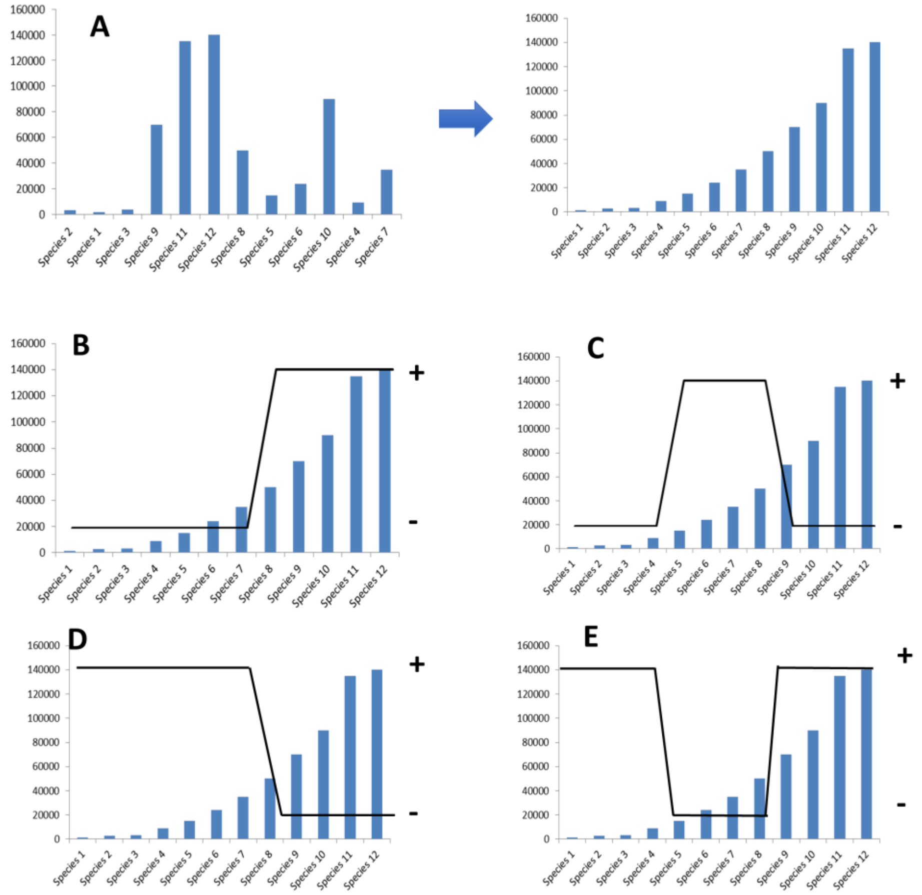

Figure S11. Visual representation of a Runs test. (A) Species (x-axis) are ranked according to the abundance of a molecular feature (y-axis), any significantly larger than expected number of runs for the same bioactivity level (i.e. bioactive extract or non-bioactive extract) with increasing molecular feature levels will result in a significant result. (B), (C), (D) and (E) Overlay of bioactivity results (+ - Bioactive extract; - - non-bioactive extract) with molecular feature levels. Note that species extracts are ranked according to molecular feature abundance. (B) – Trend expected for bioactive feature in which the activity is not hampered by increasing concentrations of feature. (C) – Trend expected for a bioactive feature in which high levels of compound could have cytotoxic effects in the disease model. (D) - Trend expected for a molecular feature that hampers bioactivity at high levels. (E) – Bioactivity observed in the higher and lower ranges of concentrations, but not at intermediate levels. This can be a special case of pattern B, in which different features may exert bioactive properties, thus when the feature is present in low amounts, the protective effect could be conferred by another compound.

6. References

1. Sumner, L.W.; Amberg, A.; Barrett, D.; Beale, M.H.; Beger, R.; Daykin, C.A.; Fan, T.W.; Fiehn, O.; Goodacre, R.; Griffin, J.L., et al. Proposed minimum reporting standards for chemical analysis Chemical Analysis Working Group (CAWG) Metabolomics Standards Initiative (MSI). *Metabolomics* **2007**, *3*, 211-221, doi:10.1007/s11306-007-0082-2.
2. Outeiro, T.F.; Lindquist, S. Yeast cells provide insight into alpha-synuclein biology and pathobiology. *Science* **2003**, *302*, 1772-1775, doi:10.1126/science.1090439.
3. Khazak, V.; Kato-Stankiewicz, J.; Tamanoi, F.; Golemis, E.A. Yeast screens for inhibitors of Ras-Raf interaction and characterization of MCP inhibitors of Ras-Raf interaction. *Methods Enzymol* **2006**, *407*, 612-629, doi:10.1016/S0076-6879(05)07048-5.
4. Araki, Y.; Wu, H.; Kitagaki, H.; Akao, T.; Takagi, H.; Shimoi, H. Ethanol stress stimulates the Ca2+-mediated calcineurin/Crz1 pathway in Saccharomyces cerevisiae. *J Biosci Bioeng* **2009**, *107*, 1-6, doi:10.1016/j.jbiosc.2008.09.005.
5. Ju, S.; Tardiff, D.F.; Han, H.; Divya, K.; Zhong, Q.; Maquat, L.E.; Bosco, D.A.; Hayward, L.J.; Brown, R.H., Jr.; Lindquist, S., et al. A yeast model of FUS/TLS-dependent cytotoxicity. *PLoS Biol* **2011**, *9*, e1001052, doi:10.1371/journal.pbio.1001052.
6. Toussaint, M.; Conconi, A. High-throughput and sensitive assay to measure yeast cell growth: a bench protocol for testing genotoxic agents. *Nat Protoc* **2006**, *1*, 1922-1928, doi:10.1038/nprot.2006.304.
7. Zhang, Y.; Butelli, E.; Alseekh, S.; Tohge, T.; Rallapalli, G.; Luo, J.; Kawar, P.G.; Hill, L.; Santino, A.; Fernie, A.R., et al. Multi-level engineering facilitates the production of phenylpropanoid compounds in tomato. *Nat Commun* **2015**, *6*, 8635, doi:10.1038/ncomms9635.
8. Butelli, E.; Titta, L.; Giorgio, M.; Mock, H.P.; Matros, A.; Peterek, S.; Schijlen, E.G.; Hall, R.D.; Bovy, A.G.; Luo, J., et al. Enrichment of tomato fruit with health-promoting anthocyanins by expression of select transcription factors. *Nat Biotechnol* **2008**, *26*, 1301-1308, doi:10.1038/nbt.1506.
9. Willingham, S.; Outeiro, T.F.; DeVit, M.J.; Lindquist, S.L.; Muchowski, P.J. Yeast genes that enhance the toxicity of a mutant huntingtin fragment or alpha-synuclein. *Science* **2003**, *302*, 1769-1772, doi:10.1126/science.1090389.
10. Bharadwaj, P.; Martins, R.; Macreadie, I. Yeast as a model for studying Alzheimer's disease. *FEMS Yeast Res* **2010**, *10*, 961-969, doi:10.1111/j.1567-1364.2010.00658.x.
11. Stathopoulos, A.M.; Cyert, M.S. Calcineurin acts through the CRZ1/TCN1-encoded transcription factor to regulate gene expression in yeast. *Genes Dev* **1997**, *11*, 3432-3444.
12. Garcia, G.; Santos, C.N.; Menezes, R. High-Throughput Yeast-Based Reporter Assay to Identify Compounds with Anti-inflammatory Potential. *Methods Mol Biol* **2016**, *1449*, 441-452, doi:10.1007/978-1-4939-3756-1_29.
13. Matheos, D.P.; Kingsbury, T.J.; Ahsan, U.S.; Cunningham, K.W. Tcn1p/Crz1p, a calcineurin-dependent transcription factor that differentially regulates gene expression in Saccharomyces cerevisiae. *Genes Dev* **1997**, *11*, 3445-3458.
